# Study of the anti-allergic and anti-inflammatory activity of *Brachychiton rupestris* and *Brachychiton discolor* leaves (Malvaceae) using in vitro models

**DOI:** 10.1186/s12906-018-2359-6

**Published:** 2018-11-09

**Authors:** Amany A. Thabet, Fadia S. Youssef, Michal Korinek, Fang-Rong Chang, Yang-Chang Wu, Bing-Hung Chen, Mohamed El-Shazly, Abdel Nasser B. Singab, Tsong-Long Hwang

**Affiliations:** 1 Department of Pharmacognosy, Faculty of Pharmacy, Ain Shams University, African Union Organization Street, Abbassia, Cairo, 11566 Egypt; 2 Graduate Institute of Natural Products, College of Pharmacy, Kaohsiung Medical University, Kaohsiung, 80708 Taiwan; 3 Department of Biotechnology, College of Life Science, Kaohsiung Medical University, Kaohsiung, 80708 Taiwan; 4 Graduate Institute of Natural Products, College of Medicine, Chang Gung University, Taoyuan, 33302 Taiwan; 5 Research Center for Chinese Herbal Medicine, Research Center for Food and Cosmetic Safety, and Graduate Institute of Health Industry Technology, College of Human Ecology, Chang Gung University of Science and Technology, Taoyuan, 33302 Taiwan; 6 National Research Institute of Chinese Medicine, Ministry of Health and Welfare, Taipei, 11221 Taiwan; 7 Research Center for Natural Products & Drug Development, Kaohsiung Medical University, Kaohsiung, 80708 Taiwan; 8 Department of Medical Research, Kaohsiung Medical University Hospital, Kaohsiung, 80708 Taiwan; 9 The Institute of Biomedical Sciences, National Sun Yat-sen University, Kaohsiung, 80424 Taiwan; 10 Department of Pharmaceutical Biology, Faculty of Pharmacy and Biotechnology, German University in Cairo, Cairo, 11835 Egypt; 11 Department of Anesthesiology, Chang Gung Memorial Hospital, Taoyuan, 33305 Taiwan; 12 Chinese Herbal Medicine Research Team, Healthy Aging Research Center, Chang Gung University, Taoyuan, 33302 Taiwan; 13 Department of Chemical Engineering, Ming Chi University of Technology, New Taipei City, 24301 Taiwan

**Keywords:** Anti-allergic, Anti-inflammatory, *Brachychiton discolor*, *Brachychiton rupestris*, Cytotoxicity, Phytochemistry

## Abstract

**Background:**

*Brachychiton rupestris* and *Brachychiton discolor* (Malvaceae) are ornamental trees native to Australia. Some members of *Brachychiton* and its highly related genus, *Sterculia*, are employed in traditional medicine for itching, dermatitis and other skin diseases. However, scientific studies on these two genera are scarce. Aiming to reveal the scientific basis of the folk medicinal use of these plants, the cytotoxicity, anti-inflammatory and anti-allergic activities of *Brachychiton rupestris* and *Brachychiton discolor* leaves extracts and fractions were evaluated. Also, phytochemical investigation of *B. rupestris* was performed to identify the compounds exerting the biological effect.

**Methods:**

Extracts as well as fractions of *Brachychiton rupestris* and *Brachychiton discolor* were tested for their cytotoxicity versus hepatoma HepG2, lung A549, and breast MDA-MB-231 cancer cell lines. Assessment of the anti-allergic activity was done using degranulation assay in RBL-2H3 mast cells. Anti-inflammatory effect was tested by measuring the suppression of superoxide anion production as well as elastase release in fMLF/CB-induced human neutrophils. Phytochemical investigation of the *n*-hexane, dichloromethane and ethyl acetate fractions of *B. rupestris* was done using different chromatographic and spectroscopic techniques.

**Results:**

The tested samples showed no cytotoxicity towards the tested cell lines. The nonpolar fractions of both *B. rupestris* and *B. discolor* showed potent anti-allergic potency by inhibiting the release of *β*-hexosaminidase. The dichloromethane fraction of both species exhibited the highest anti-inflammatory activity by suppressing superoxide anion generation and elastase release with IC_50_ values of 2.99 and 1.98 μg/mL, respectively for *B. rupestris*, and 0.78 and 1.57 μg/mL, respectively for *B. discolor.* Phytochemical investigation of various fractions of *B. rupestris* resulted in the isolation of *β*-amyrin acetate (1), *β*-sitosterol (2) and stigmasterol (3) from the *n*-hexane fraction. Scopoletin (4) and *β*-sitosterol-3-*O*-*β*-D-glucoside (5) were obtained from the dichloromethane fraction. Dihydrodehydrodiconiferyl alcohol 4-*O*-*β*-D-glucoside (6) and dihydrodehydrodiconiferyl alcohol 9-*O*-*β*-D-glucoside (7) were separated from the ethyl acetate fraction. Scopoletin (4) showed anti-allergic and anti-inflammatory activity.

**Conclusions:**

It was concluded that the nonpolar fractions of both *Brachychiton* species exhibited anti-allergic and anti-inflammatory activities.

**Electronic supplementary material:**

The online version of this article (10.1186/s12906-018-2359-6) contains supplementary material, which is available to authorized users.

## Background

Allergy is one of the most popular diseases worldwide and its great prevalence makes allergic disorder a growing global concern [1]. Allergic reaction can be defined as the development of signs and symptoms of hypersensitivity reactions upon exposure to certain allergenic substances resulting in massive production of allergen-specific IgE and allergen-specific T-cell populations [2]. Allergic reaction can be a life-threatening condition especially in anaphylaxis and severe asthma or it can be a chronic condition that interferes with the quality of life such as in eczema and allergic rhinitis [3].

Inflammation is another common disorder which is an innate immune response from the host defense mechanism. It consists of a series of complex biological processes aiming to combat infection and tissue injury. These processes lead to accumulation of plasma and blood cells in the tissue in addition to the release of inflammatory mediators aiming to reestablish tissue structures and function [4, 5]. Untreated inflammation can lead to a chronic condition which is characterized as a very long-term inflammation affecting the remodeling of tissue for many weeks and even years. It is considered as a main cause in the development of a various life threatening disorders, such as neurodegenerative diseases and cancers [4].

Non-steroidal anti-inflammatory drugs (NSAIDs) constitute the commonly adopted classes for the alleviation of inflammation and related conditions. Meanwhile, their intolerable side effects represented by gastrointestinal ulcers, and perforation with concomitant bleeding are the main obstacles facing their therapeutic usage [6]. On the contrary, nature continues to serve as a rich and appealing source of novel, safer, and cheaper bioactive molecules in comparison to many synthetic drugs. A plethora of plant extracts, as well as isolated compounds, possess notable anti-allergic and anti-inflammatory activities, as previously reported [5, 7–11].

Malvaceae, the mallows, is a family that comprises more than 200 genera and 2300 species. A great diversity of phytoconstituents such as triterpenes, flavonoids, coumarins, as well as alkaloids was previously reported in the members of this family [12, 13]. *Brachychiton* (Malvaceae) is a small genus native to Australia comprising of 30 species [14, 15]. Recently, *Brachychiton* has been considered as a separate genus from *Sterculia* as proved by the detailed investigation of its follicles, seed coats and embryo [14]. Members of the *Brachychiton* genus were used as food by Australian Aborigines and some are used as ornamental trees or shrubs [16, 17]. Different members of the genus possess several interesting biological effects such as antioxidant, antibacterial, anti-hyperglycemic, hepatoprotective and anti-schistosomal activities [18–21]. Phytochemical studies of various members of *Brachychiton* sp. resulted in the identification of various classes of compounds such as flavonoids, coumarins, triterpenes, sterols, and alkaloids [22–24]. *Brachychiton rupestris* is commonly known as “Queensland bottle tree” because it is native to Queensland and has a bottle shaped trunk. *B. discolor* (synonym *B. luridus*) is commonly called the lacebark tree [25–27]. The mucilage and ethyl acetate fraction of *B. rupestris* leaves were previously investigated for their in vivo anti-hyperglycemic effect and the phytochemical investigation of this species led to the isolation and identification of flavonoid aglycones and glycosides from the leaves [20, 28]. However, no complete phytochemical study was done on this species. Regarding *B. discolor*, two complete phytochemical studies were reported on this species where many classes of compounds were reported from the leaves, seeds and roots including triterpenes, flavonoids, phenolic acids, coumarins and alkaloids [23, 24].

Tracing current literature, nothing was found regarding the anti-allergic and anti-inflammatory effects of *B. rupestris*. However, different studies were carried out confirming the anti-allergic and anti-inflammatory activities of several triterpenes such as *β*-amyrin, oleanolic acid and lupeol [29–33] which were also isolated from *B. discolor* [23, 24]. Another species (*B. populneus*) was reported to be effective in relieving pain and skin diseases in folk medicine [34]. Furthermore, many members of the related genus, *Sterculia*, are popular in folk medicine for alleviating itching, dermatitis, boils, inflammations and other skin diseases [35–40]. Herein, we investigated the anti-allergic and anti-inflammatory activities of the methanol extracts and fractions of both *B. rupestris* and *B. discolor* leaves. The cytotoxic effect of *B. rupestris* and *B. discolor* leaves extracts and fractions was also evaluated to ascertain their safety. Additionally, the isolation and structural elucidation of the major constituents from the bioactive fractions of *B. rupestris* was achieved within this work.

## Methods

### Plant materials

The leaves of *B. rupestris* (T.Mitch.ex Lindl) K.Schum and *B. discolor* F.Muell were obtained from El-Orman Botanical Garden, Giza, Egypt, in summer 2014. The plants were generously authenticated by Prof. Dr. Mohamed El-Gibaly, Department of Botany, National Research Center (NRC), Giza, Egypt. Voucher specimens (PHG-P-BR-248 and PHG-P-BL-249) for *B. rupestris* and *B. discolor* (*B. luridus*), respectively were kept at the Pharmacognosy Department, Faculty of Pharmacy, Ain Shams University.

### Extraction and fractionation

Total amount of 3.05 kg of *B. rupestris* air-dried leave were crushed, macerated in 29 L of distilled methanol for three times and filtered. Subsequently, the obtained filtrate was evaporated in vacuo at low temperature (45 °C) till dryness and then subjected to lyophilization to give 333.56 g of the total methanol extract. A portion of the extract (300 g) was successively partitioned with *n*-hexane (37.9 L), dichloromethane (4.8 L) and ethyl acetate (5.2 L) to give 54.35, 8.54 and 5.91 g, respectively along with the remaining hydromethanolic fraction estimated as 191.5 g.

Similarly, for *B. discolor*, the crushed air-dried leaves 600 g were macerated in distilled methanol (6 L × 3), filtered, and evaporated at 45 °C under reduced pressure till dryness to yield 36 g of the total methanol extract. Then, 11 g of the total extract were fractionated using 430 mL of *n*-hexane, 300 mL of dichloromethane and 300 mL of ethyl acetate successively to give 1.3, 1.2, and 0.9 g of the dried residues, respectively.

### Biological investigations

#### In vitro assessment of the cytotoxic activity

##### Cell culture

The cytotoxicity of *B. rupestris* and *B. discolor* total extracts as well as their obtained fractions was examined on A549 (adenocarcinoma human alveolar basal epithelial cells), HepG2 (human liver cancer cell line) and MDA-MB-231 (invasive ductal carcinoma) cells. Cells were preserved in Dulbecco’s modified Eagle’s medium-high glucose powder (DMEM) containing 10% heat-inactivated fetal bovine serum (FBS), 1 mM sodium pyruvate, 100 μg/mL streptomycin, 100 U/mL penicillin, and 2 mM L-glutamine. Cells were cultured in culture dishes (Cellstar) that were kept in a humidified chamber supplied with 5% (*v*/v) CO_2_ at 37 °C. Then the cells were maintained as a monolayer culture adopting serial subculturing. Cells growing in the logarithmic phase were employed in all experiments [41].

##### Cytotoxicity assay

MTT (methylthiazoltetrazolium) assay was employed to evaluate the cytotoxic activity of the tested samples against human cancer cells [42, 43]. Trypsinized cell suspensions were freshly prepared and then planted in a 96-well culture plate followed by overnight incubation. Tested samples were prepared in dimethyl sulfoxide (DMSO) to form stock solutions of 1 mg/mL. Cells were treated with the tested samples using different concentrations (2.5–20 μg/mL) then incubated for 72 h at 37 °C under 5% CO_2_. After the incubation, and removal of the cells medium, 100 μL of MTT solution was added to each well followed by incubation of the cells for 1 h. The formed formazan crystals were dissolved in DMSO after the removal of the medium to measure absorbance at 550 nm. The percentage of cell viability was calculated by the following formula:

$$ \%\mathrm{cell}\ \mathrm{viability}=\frac{\mathrm{O}.\mathrm{D}\ \mathrm{of}\ \mathrm{treated}\ \mathrm{cells}-\mathrm{O}.\mathrm{D}\ \mathrm{of}\ \mathrm{culture}\ \mathrm{medium}}{\mathrm{O}.\mathrm{D}\ \mathrm{of}\ \mathrm{untreated}\ \mathrm{cells}-\mathrm{O}.\mathrm{D}\ \mathrm{of}\ \mathrm{culture}\ \mathrm{medium}}\times 100 $$

Where O.D = optical density

Cytotoxicity was expressed as % cell inhibition. Doxorubicin was used as the positive control.

#### In vitro assessment of the anti-allergic activity

##### Chemicals and reagents

DMEM, dexamethasone*, p*-nitrophenyl-*N*-acetyl-D-glucosaminide (*p*-NAG), MTT (3-(4,5-dimethylthiazol-2-yl)-2,5-diphenyltetrazolium bromide), penicillin and streptomycin, calcium ionophore A23187, mouse anti-DNP (dinitrophenyl) IgE antibody, and DMSO were purchased from Sigma-Aldrich (St. Louis, MO, USA). Moreover, FBS was obtained from Hyclone (Logan, UT, USA). Dinitrophenyl-conjugated bovine serum albumin (DNP-BSA) was purchased from Merck (Kenilworth, NJ, USA). Additional chemicals as well as reagents were purchased at the highest possible purity.

##### Cell culture

The mucosal mast cell-derived rat basophilic leukemia (RBL-2H3) cell line was obtained from the American Type Culture Collection. Cells were grown in DMEM medium accompanied with 10% FBS in addition to 100 U/mL penicillin plus 100 μg/mL streptomycin. Cells were cultured in 10 cm cell culture dishes (Cellstar) at 37 °C with 5% CO_2_ in air.

##### Cell viability assay

MTT assay was used to assess the toxic effects of samples on RBL-2H3 cells [44] and was done as previously mentioned [42, 43]. All experiments were done in triplicates. DMSO served as the negative control not affecting the growth of RBL-2H3 cells. Triton X-100 (0.5% solution) was employed as the positive control resulting in the death of all cells in a well.

##### Degranulation β-hexosaminidase assay induced by A23187

A23187-induced degranulation in RBL-2H3 cells was evaluated by a *β*-hexosaminidase activity assay as previously reported employing certain modifications [45, 46]. RBL-2H3 cells were seeded into 96-wells plate using a density of 2 × 10^4^ cells/well and were incubated at 37 °C for 5 h in 5% CO_2_. Cells were washed with PBS (phosphate buffered saline) and then various concentrations of samples or medium (untreated control) were added to each well (100 μL), and the treated cells were incubated at 37 °C in 5% CO_2_ for 20 h. The cells were stimulated by calcium ionophore A23187 (1 μM) diluted in Tyrode’s buffer (135 mM NaCl, 1.8 mM CaCl_2_, 5 mM KCl, 1.0 mM MgCl_2_, 5.6 mM glucose, 20 mM HEPES at pH 7.4), and kept at 37 °C in 5% CO_2_ for 1 h. For the total amount of *β*-hexosaminidase release, the unstimulated cells were lysed using 0.5% Triton X-100. Untreated unstimulated cells represented spontaneous *β*-hexosaminidase release. The control wells were represented by the stimulated untreated cells. The cells supernatants (50 μL) were incubated with equal volume of 1 μM of p-NAG (50 μL), a substrate for *β*-hexosaminidase, prepared in 0.05 M citrate buffer (pH 4.5) for 1 h at 37 °C. The reaction was stopped by 100 μL of stop buffer (0.1 M Na_2_/NaHCO_3_, pH 10.0). Microplate reader was used to measure the absorbance at 405 nm. The inhibition percentage of *β*-hexosaminidase release from RBL-2H3 cells was calculated using the following equation:

$$ \mathrm{Inhibition}\ \left(\%\right)=\left[1-\frac{\left(\mathrm{ODsample}-\mathrm{ODspontaneous}\right)}{\left(\mathrm{ODcontrol}-\mathrm{ODspontaneous}\right)\ }\right]\times 100 $$

Dexamethasone (10 nM) was employed as the positive control.

##### Degranulation β-hexosaminidase assay induced by IgE

*β*-Hexosaminidase release from the activated RBL-2H3 cells was measured as previously reported [45, 47], with some modifications. The inhibition percentage of antigen-induced *β*-hexosaminidase release from RBL-2H3 cells was assessed in a similar way as described above in the degranulation A23187-induced *β*-hexosaminidase assay, except of the stimulation process. The cells were sensitized with anti-DNP IgE (0.1 μg/mL) for at least 2 h and then washed with pre-warmed Tyrode’s buffer, followed by stimulation by antigen DNP-BSA (100 ng/mL). Dexamethasone (10 nM) was employed as the positive control.

#### In vitro assessment of the anti-inflammatory activity

##### Preparation of human neutrophils

Blood was withdrawn from 20 to 35 years old healthy human donors adopting a protocol approved by the institutional review board at Chang Gung Memorial Hospital. Isolation of neutrophils was done employing a standard method which was previously reported [48].

### Measurement of superoxide generation

Ferricytochrome *c* (0.5 mg/mL) and Ca^2^ (1 mM) were incubated with neutrophils at 37 °C for 2 min, followed by the treatment with the tested samples for 5 min. Cells activation was done using formyl-methionyl-leucyl-phenylalanine (fMLF, 100 nM)/cytochalasin B (CB, 1 μg/mL) for 10 min. The absorbance was detected at 550 nm in a double-beam spectrophotometer Hitachi U-3010. Superoxide inhibition was determined by lowering ferricytochrome *c* as reported previously [48, 49]. The differences in absorbance between the measurements in the presence of superoxide (100 U/mL) and its absence divided by the extinction coefficient for the reduction of ferricytochrome *c* (*ε* = 21.1/mM/10 mm) were used as the basis for calculations. Genistein was adopted as the positive control [50, 51].

#### Measurement of elastase release

The release of elastase was determined by assessing the degranulation of azurophilic granules [48, 49]. An elastase substrate MeO-Suc-Ala-Ala-Pro-Val-*p*-nitroanilide (100 μM) was equilibrated with neutrophils at 37 °C for 2 min, followed by incubation with drugs for 5 min. Activation of the cells was done using 100 nM fMLF and 0.5 μg/mL CB, and then the variations in absorbance were detected at 405 nm. The results are shown as the percentage of the initial rate of elastase release in the fMLF/CB-activated, drug-free control system. Genistein was employed as the positive control [50, 51].

#### Statistical analysis

Results are represented as mean ± SD value of at least three independent measurements unless otherwise specified. The 50% inhibitory concentration (IC_50_) was determined using the dose-response curve which was constructed by plotting the percentage of inhibition versus concentrations (linear function, Microsoft Office). Statistical analysis was done using one-way analysis of variance (ANOVA) followed by Dunnet’s test (GraphPad Prism 6.0, GraphPad Software, Sand Diego, CA, USA, anti-allergic assay) or Student’s *t*-test (Sigma Plot, Systat software, Systat Software Inc., San Jose, CA, USA, anti-inflammatory assay). Values which show *p < 0.05, **p < 0.001 are statistically significant.

### Phytochemical investigations

#### General experimental procedures

^1^H and ^13^C (APT) NMR analyses were done using a Bruker Ascend 400/R spectrometer (Burker Avance III, Fallanden Switzerland) at the Center for Drug Discovery, Research and Development, Faculty of Pharmacy, Ain Shams University using 400 and 100 MHz the operating frequencies. Chemical shifts were reported in *δ* ppm and were related to that of the solvents. Dissolution of the tested samples was done using various deuterated solvents (Sigma Aldrich, Germany) in 3 mm NMR tubes (Bruker). Spectra were recorded at 25 °C; *δ* ppm relative to tetramethylsilane (Me_4_Si) as the internal standard. Two-dimensional (2D) NMR experiments (^1^H, ^1^H-^1^H COSY; ^1^H-^13^C HSQC; ^1^H-^13^C HMBC) were done using the pulse sequences from the Bruker user library. Waters Xevo TQD mass spectrometer supplied with UPLC Acquity mode (*Milford*, USA) was employed to carry out ESI-MS analysis. Normal phase column chromatography was done using silica gel (Kieselgel 60, 70–230, and 230–400 mesh, Merck KGaA, Darmstadt, Germany). TLC analysis was done utilizing normal phase silica gel precoated plates F_254_ (Merck, Germany). Detection of TLC spots was done using UV light at 254 nm and 365 nm as well as by spraying with 10% H_2_SO_4_ with subsequent heating on a hot plate at 100 °C.

#### Isolation of the secondary metabolites from the bioactive fractions

**Fig. 1:**
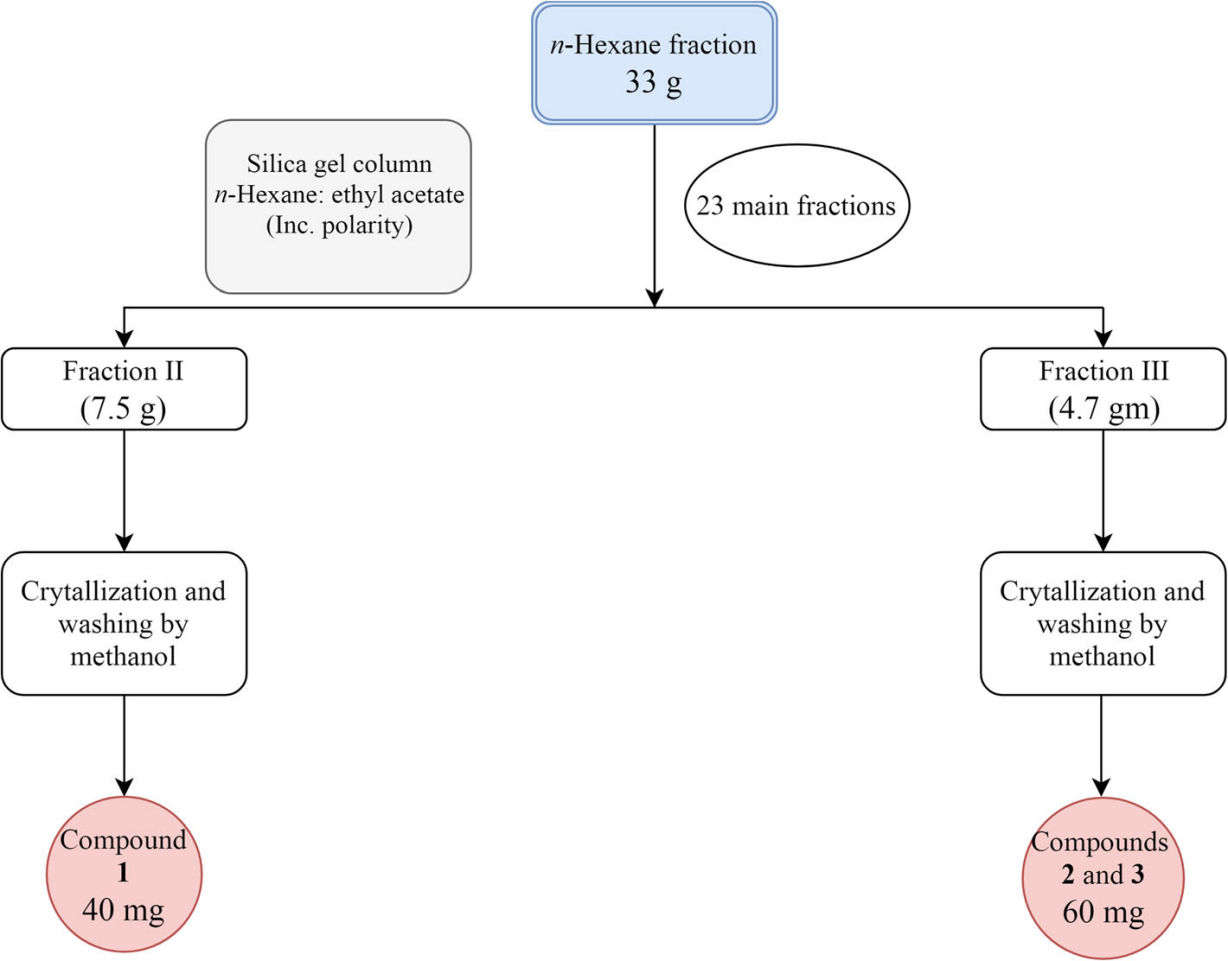
Scheme showing the chromatographic fractionation of the *n*-hexane fraction

The *n-*hexane fraction (33 g) of *B. rupestris* was chromatographed on silica gel (600 g) employing *n*-hexane:EtOAc with increasing polarity to give 23 major fractions. Fraction II was further eluted with a mixture of *n*-hexane: EtOAc (9.0:1.0) from which compound **1** (40 mg) was precipitated as a white amorphous powder. A mixture of compounds **2** and **3** (60 mg) was precipitated from fraction III as white crystalline needles using the solvent system *n*-hexane:EtOAc (9.0:1.0) as illustrated in Fig. 1.

**Fig. 2:**
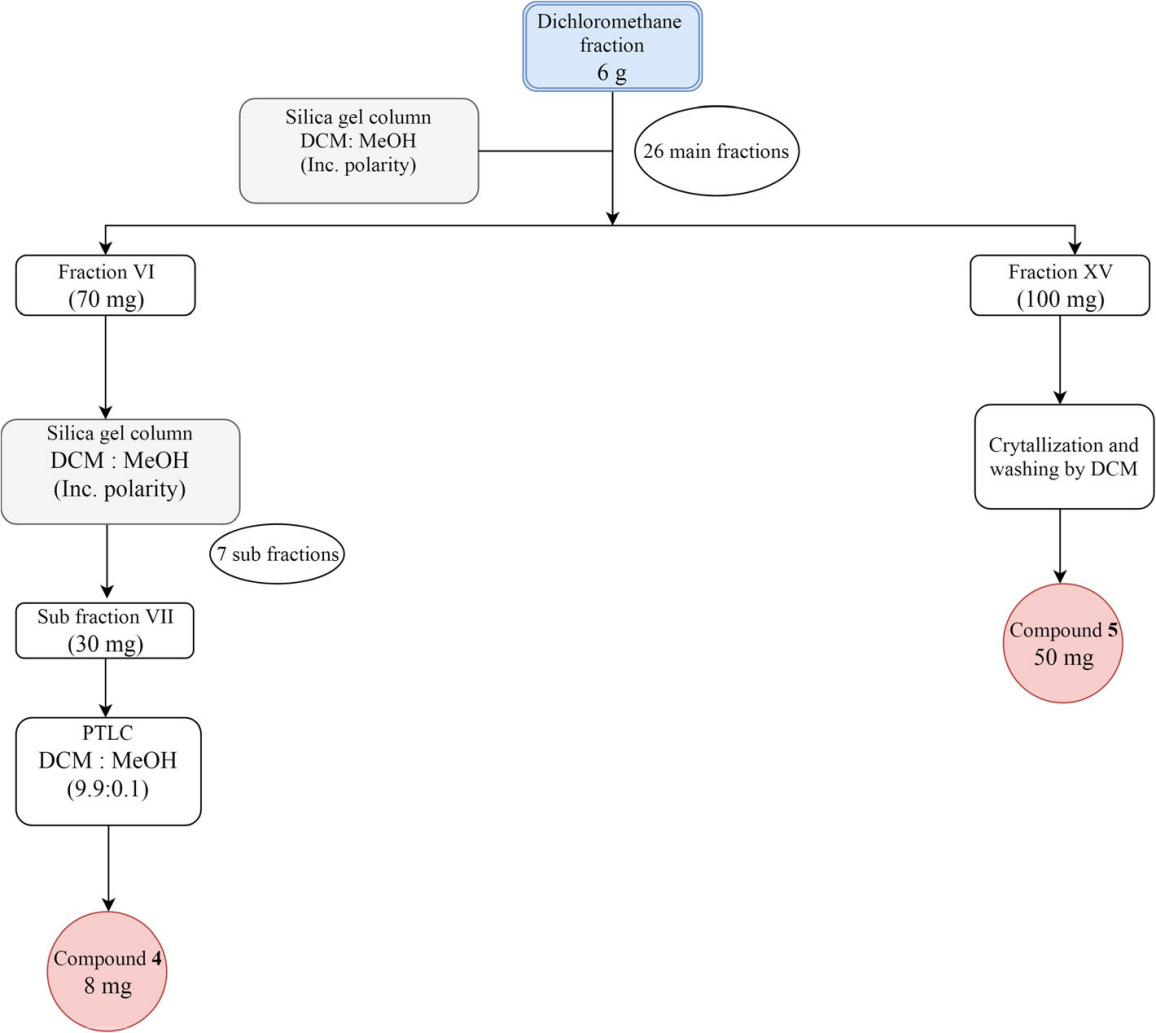
Scheme showing the chromatographic fractionation of the dichloromethane fraction

The dichloromethane fraction of *B. rupestris* (6 g) was chromatographed on silica gel (300 g) using mixtures of CH_2_Cl_2_:CH_3_OH with increasing polarity as eluents to afford 26 major fractions. Fraction VI (70 mg) was further eluted with dichloromethane and was subjected to silica gel column using a mixture of CH_2_Cl_2_:CH_3_OH to give seven subfractions. Subfraction **7** (30 mg) was eluted with a mixture of CH_2_Cl_2_:CH_3_OH (9.9:0.1) and purified over preparative TLC which resulted in the separation of compound **4** (8 mg) that showed strong fluorescent yellow color. Fraction XV was eluted using a mixture of CH_2_Cl_2_:CH_3_OH (9.6:0.4) from which compound **5** (50 mg) was precipitated as a yellow powder as shown in Fig. 2.

**Fig. 3:**
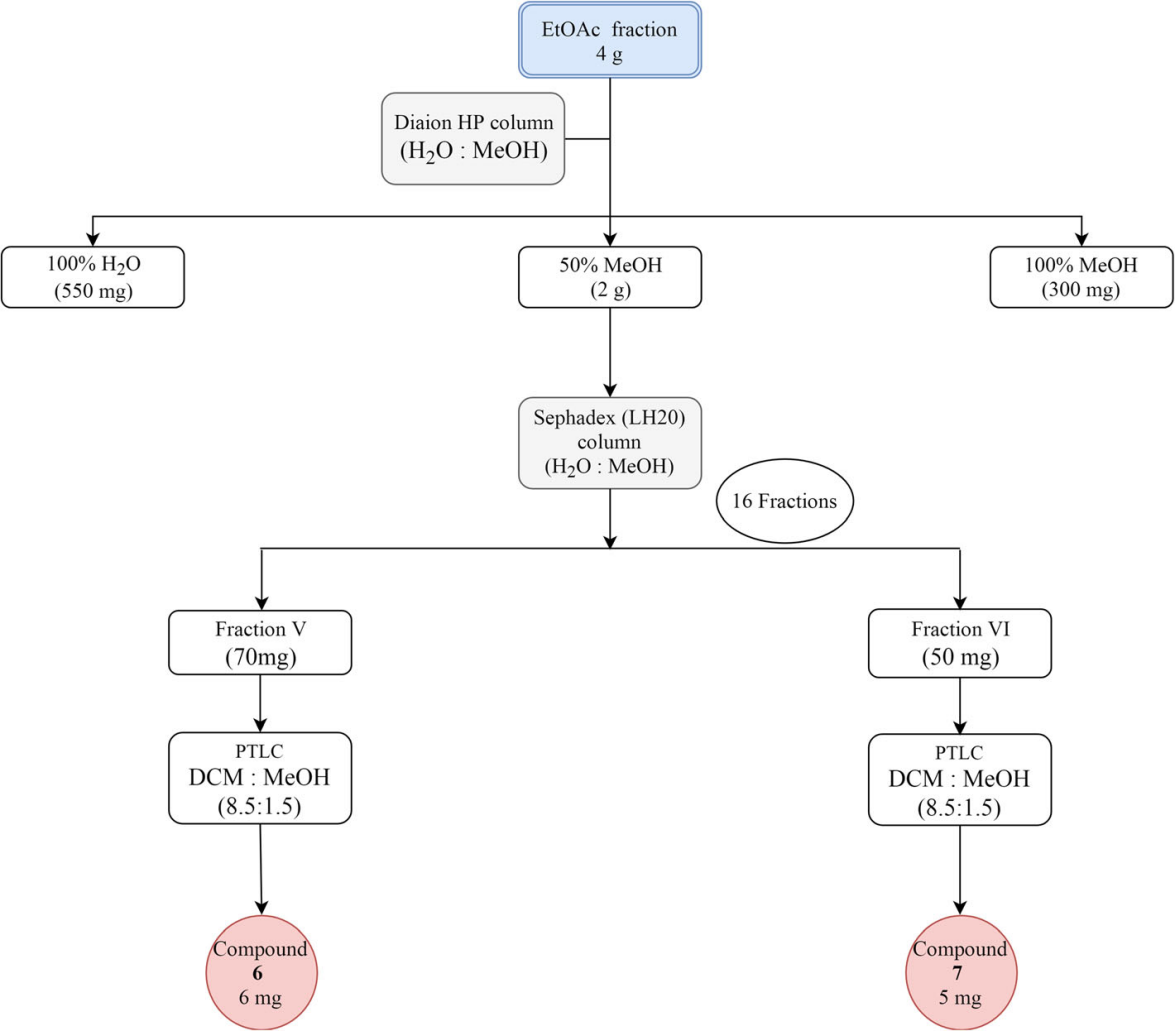
Scheme showing the chromatographic fractionation of the ethyl acetate fraction

The EtOAc fraction (4 g) was applied on the top of 150 g Diaion HP column using water, 50% methanol, 100% methanol as the mobile phases. The 50% methanol fraction (2 g) was the most promising fraction after comparing its TLC with the other fractions and was applied on the top of 40 g Sephadex® LH 20 and eluted using water and methanol of decreasing polarity to give 16 fractions. Fraction V (70 mg) and fraction VI (50 mg) were eluted using water 100% and were purified over preparative TLC using CH_2_Cl_2_:CH_3_OH (8.5:1.5) as the mobile phase to separate compounds **6** (6 mg) and **7** (5 mg), respectively (Fig. 3).

#### Spectroscopic data of compounds 1–7

*β*-Amyrin acetate (**1**)

It was isolated as a white amorphous powder; with R_*f*_ = 0.530 in *n*-hexane:EtOAc (9.5:0.5). ^1^H NMR (400 MHz, CDCl_3_), ^13^C NMR (100 MHz, CDCl_3_) and 2D NMR spectroscopic data are displayed in the Additional file 1: Figure S1).

*β*-Sitosterol (**2**) and Stigmasterol (**3**)

They were isolated as white crystalline needles; showing R_*f*_ = 0.206 in *n*-hexane:EtOAc (9:1). ^1^H-NMR (400 MHz, CDCl_3_), ^13^C NMR (100 MHz, CDCl_3_) and 2D NMR spectral data are displayed in the Additional file 1: Figure S2.

Scopoletin (**4**)

It was obtained as a yellow powder; with R_*f*_ = 0.630 in CH_2_Cl_2_:CH_3_OH (9.9:0.1). ^1^H-NMR (400 MHz, CD_3_OD) (*δ* ppm): 7.75 (1H, *d*, *J* = 9.1, H-4), 6.80 (1H, *s*, H-5), 6.45 (1H, *s*, H-8), 5.85 (1H, *d*, *J* = 9.1 Hz, H-3), 3.81 (3H, *s*, 6-OCH_3_). ^13^C NMR data (100 MHz, CD_3_OD) (*δ* ppm): 166.26 (C-2), 153.86 (C-7), 151.4 (C-6), 146.99 (C-4), 107.65 (C-5), 105.50 (C-3), 104.59, (C-8), 56.03 (6-OCH_3_). It exhibited a deprotonated molecular ion peak at *m/z* 190.8 [M-H]^−^ in ESI-MS negative ion mode, corresponding to the molecular formula C_10_H_8_O_4_ (Additional file 1: Figure S3).

*β*-Sitosterol-3-O-*β*-D-glucoside (**5**)

It was isolated as a buff amorphous powder; with R_*f*_ = 0.630 in CH_2_Cl_2_:CH_3_OH (9.2:0.8). ^1^H-NMR (400 MHz, DMSO-*d*_*6*_), ^13^C NMR (100 MHz, DMSO-*d*_*6*_) and 2D NMR spectroscopic data are displayed in the Additional file 1: Figure S4.

Dihydrodehydrodiconiferyl alcohol 4-*O*-*β*-D-glucoside (**6**)

It was obtained as a yellowish white amorphous powder; with R_*f*_ = 0.259 in CH_2_Cl_2_:CH_3_OH (8.5:1.5). ^1^H-NMR (400 MHz, CD_3_OD), ^13^C NMR data (100 MHz, CD_3_OD) are illustrated in Table 4, (Additional file 1: Figure S5).

Dihydrodehydrodiconiferyl alcohol 9-*O*-*β*-D-glucoside (**7**)

It was obtained as a yellowish white amorphous powder; with R_*f*_ = 0.304 in CH_2_Cl_2_:CH_3_OH (8.5:1.5). ^1^H-NMR (400 MHz, CD_3_OD), ^13^C NMR (100 MHz, CD_3_OD) and 2D NMR spectroscopic data are displayed in Table 4 and the Additional file 1: Figure S6.

## Results

### In vitro assessment of the cytotoxic activity of *B. rupestris* and *B. discolor*

The cytotoxicity of the total methanol extracts and fractions of both *B. rupestris* and *B. discolor* was evaluated versus HepG2, A549 and MDA-MB-231 cancer cells using doxorubicin as the positive control. Extracts and fractions of both species at 20 μg/mL exhibited no cytotoxic activity against any of the tested cell lines. Noteworthy to mention that doxorubicin showed 91.28, 97.69 and 98.05% cell growth inhibition against HepG2, MDA-MB-231 and A549, respectively at 2 μg/mL. The results are illustrated in Table 1. Together with the nontoxic effects of all samples towards RBL-2H3 mast cells (see the following section, and Table 2) the results suggested that both species extracts and fractions exhibited no cytotoxicity against the tested cancer cell lines*.*

**Table 1:** In vitro cytotoxicity of different extracts and fractions of *B. rupestris* and *B. discolor* against HepG2, MDA-MB-231 and A549 cell lines

Cell line	BRT	BRH	BRD	BRE	BRR	BDT	BDH	BDD	BDE	doxorubicin
HepG2	1.28	−2.078	−8.02	−8.43	−13.74	1.66	−7.43	−16.18	−6.62	91.28 ± 0.3
MDA-MB-231	−15.86	− 9.70	−18.83	−25.52	− 23.30	− 6.19	− 6.73	13.17	−20.64	97.69 ± 0.4
A549	0.44	−0.81	7.85	−8.37	−0.89	12.59	8.18	14.99	−6.52	98.05 ± 0.0

Results are presented as growth inhibition percentage at concentration of 20 μg/mL, mean (*n* = 1). Doxorubicin (2 μg/mL) was used as the reference drug, mean ± SD (*n* = 2). BRT: *B. rupestris* total methanol extract; BHT: *B. rupestris n-*hexane fraction; BRD: *B. rupestris* dichloromethane fraction; BRE: *B. rupestris* ethyl acetate fraction; BRR: *B. rupestris* remaining MeOH(aq) fraction; BDT: *B. discolor* total methanol extract; BDH: *B. discolor n-*hexane fraction; BDD: *B. discolor* dichloromethane fraction; BDE: *B. discolor* ethyl acetate fraction

**Table 2:** Anti-allergic activity of *B. rupestris* and *B. discolor* extracts and fractions

Sample	% viability, RBL-2H3^a^	% inhibition of A23187-induced *β*-hexosaminidase release^b^	
	100 μg/mL	10 μg/mL	100 μg/mL
BRT	99.0 ± 1.7	3.0 ± 5.2	25.7 ± 2.1^**^
BRH	96.7 ± 4.0	3.3 ± 5.8	39.0 ± 13.1^**^
BRD	95.3 ± 8.1	4.3 ± 7.5	19.0 ± 4.4^*^
BRE	97.7 ± 4.0	2.0 ± 3.5	7.0 ± 5.2
BRR	99.0 ± 1.7	4.3 ± 5.1	3.7 ± 6.4
BDT	99.0 ± 1.7	3.7 ± 6.4	16.0 ± 5.0^*^
BDH	99.7 ± 0.6	4.3 ± 7.5	30.3 ± 3.1^**^
BDD	100.0 ± 0.0	0.0 ± 0.0	44.0 ± 7.8^**^
BDE	100.0 ± 0.0	1.7 ± 2.9	0.3 ± 0.6

^a^The cytotoxicity of samples towards RBL-2H3 cells was evaluated using MTT viability assay and none of the samples showed any toxicity; results are presented as mean ± SD (*n* = 3)

^b^Dexamethasone (10 nM) was used as the positive control and inhibited 62.0 ± 9.5%^**^ of A23187-induced *β*-hexosaminidase release in RBL-2H3 cells. Results are presented as mean ± SD (*n* = 3); *p < 0.05, ^**^p < 0.001 compared with the control value (A23187 only)

BRT *B. rupestris* total methanol extract, BRH *B. rupestris n*-hexane fraction, BRD *B. rupestris* dichloromethane fraction, BRE *B. rupestris* ethyl acetate fraction, BRR *B. rupestris* remaining MeOH(aq) fraction, BDT *B. discolor* total methanol extract, BDH *B. discolor n*-hexane fraction, BDD *B. discolor* dichloromethane fraction, BDE *B. discolor* ethyl acetate fraction

### In vitro assessment of the anti-allergic activity of *B. rupestris* and *B. discolor*

The anti-allergic activity of the total methanol extracts and fractions of both *B. rupestris* and *B. discolor* was assessed using degranulation assay in RBL-2H3 mast cell model and the results are presented in Table 2. Initially, the cytotoxic effect of all samples was tested against RBL-2H3 cells using MTT viability assay. All samples were found to be nontoxic at 100 μg/mL. The samples were subjected to the anti-allergic assay by evaluating their inhibitory effect on *β*-hexosaminidase release in RBL-2H3 cells induced by calcium ionophore, A23187. According to our results, *B. rupestris* and *B. discolor* crude methanol extracts (BRT 25.7% and BDT 16.0% inhibition) and nonpolar *n*-hexane (BRH 39.0%, BDH 30.3% inhibition) and dichloromethane fractions (BRD 19.0%; and BDD 44.0% inhibition) exhibited significant inhibition of *β*-hexosaminidase release in A23187-induced degranulation assay at 100 μg/mL (Table 2). Dexamethasone, a positive control, showed 62.0% inhibition of *β*-hexosaminidase release at 10 nM.

### In vitro assessment of the anti-inflammatory activity of *B. rupestris* and *B. discolor*

Similarly, the anti-inflammatory activity was determined for the total methanol extracts and fractions of *B. rupestris* and *B. discolor* and the results are presented in Table 3. Both *Brachychiton* species exhibited a promising inhibitory activity on superoxide anion production as well as elastase release in fMLF-activated human neutrophils indicating their potential applications for the alleviation of both acute and chronic inflammatory disorders. All samples inhibited superoxide anion generation showing IC_50_ values between 0.78 and 6.25 μg/mL in addition to inhibition of elastase release showing IC_50_ values ranging from 1.57 to > 10 μg/mL (Table 3). The most potent fractions, dichloromethane fractions of *B. rupestris* (BRD) and *B. discolor* (BDD) inhibited superoxide anion generation with IC_50_ values 2.99 μg/mL (BRD) and 0.78 μg/mL (BDD), and inhibited elastase release with IC_50_ values 1.98 μg/mL (BRD) and 1.57 μg/mL (BDD). Such activities indicated comparable or even better inhibitory potential than that of genistein (superoxide IC_50_ 0.41 μg/mL and elastase IC_50_ 4.41 μg/mL), a known anti-inflammatory natural product [50, 51]. The dichloromethane fractions (BRD and BDD) were capable of almost completely abolishing oxidative burst and degranulation in fMLF-activated human neutrophils at 10 μg/mL (data not shown). Meanwhile, the ethyl acetate fraction of both species showed anti-inflammatory activity by inhibiting elastase release showing IC_50_ values of 2.71 μg/mL for *B. rupestris* (BRE) and 2.95 μg/mL for *B. discolor* (BDE).

**Table 3:** Effect of the total extracts and fractions of *B. rupestris* and *B. discolor* on superoxide anion generation and elastase release in fMLF/CB-induced human neutrophils

Sample	Superoxide anion generation^a^	Elastase release^a^
	IC_50_ (μg/mL)^b^	IC_50_ (μg/mL)^b^
BRT	4.92 ± 1.47	3.82 ± 0.55
BRH	5.69 ± 0.80	3.73 ± 1.16
BRD	2.99 ± 0.73	1.98 ± 1.54
BRE	6.25 ± 3.10	2.71 ± 0.79
BRR	3.01 ± 1.91	> 10^c^
BDT	4.73 ± 0.97	5.37 ± 1.23
BDH	6.25 ± 2.18	6.04 ± 2.32
BDD	0.78 ± 0.29	1.57 ± 0.84
BDE	5.22 ± 1.35	2.95 ± 1.08
genistein	0.41 ± 0.09	4.41 ± 1.99

^a^IC_50_ values, results are presented as mean ± SD (*n* = 3), compared with the control value (formyl-methionyl-leucyl-phenylalanine/cytochalasin B, fMLF/CB)

^b^Concentration necessary for 50% inhibition (IC_50_)

^c^BRR exerted significant inhibitory activity in superoxide anion generation (49.6 ± 2.9%, **p < 0.001) at 10 μg/mL. BRT *B. rupestris* total methanol extract, BRH *B. rupestris n*-hexane fraction, BRD *B. rupestris* dichloromethane fraction, BRE *B. rupestris* ethyl acetate fraction, BRR *B. rupestris* remaining MeOH(aq) fraction, BDT *B. discolor* total methanol extract, BDH *B. discolor n*-hexane fraction, BDD *B. discolor* dichloromethane fraction, BDE *B. discolor* ethyl acetate fraction

### Phytochemical investigations

**Fig. 4:**
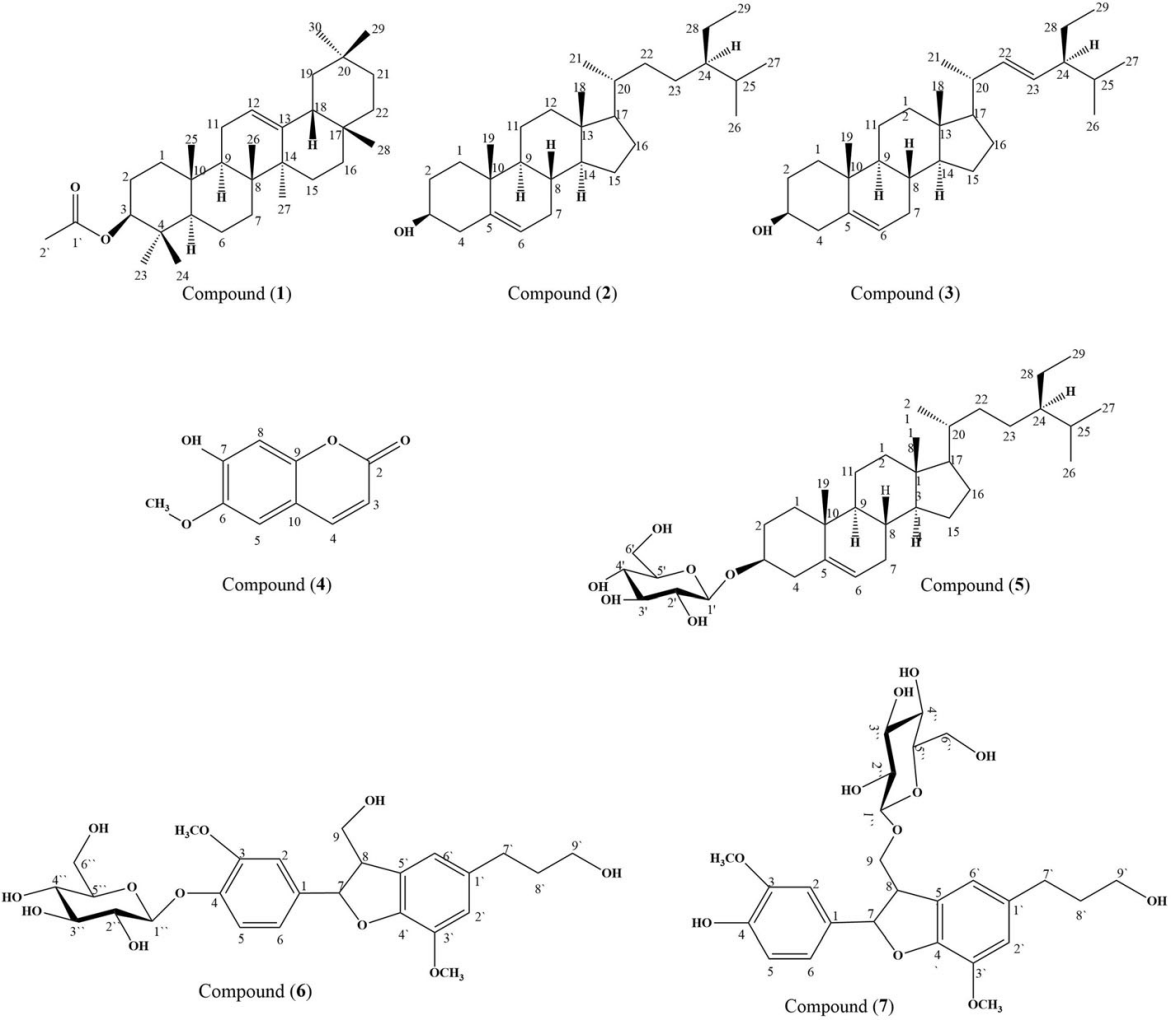
Structures of identified compounds from *n*-hexane, dichloromethane and ethyl acetate fractions of *B. rupestris*

In-depth phytochemical investigation was performed on the most bioactive fractions of *B. rupestris* leaves including the *n*-hexane, the dichloromethane and ethyl acetate fractions that showed the highest anti-allergic and anti-inflammatory activities. Three compounds were isolated and structurally elucidated from the *n*-hexane fraction which were *β*-amyrin acetate (**1**) [29], *β*-sitosterol (**2**) [52], stigmasterol (**3**) [52]. Meanwhile, two compounds were obtained from the dichloromethane fraction including scopoletin (**4**) [53, 54] and *β*-sitosterol-3-O-*β*-D-glucoside (**5**) [55] which were isolated for the first time from *B. rupestris* leaves. Furthermore, two neolignans were obtained from the ethyl acetate fraction, dihydrodehydrodiconiferyl alcohol 4-*O*-*β*-D-glucoside (**6**) [56] and dihydrodehydrodiconiferyl alcohol 9-*O*-*β*-D-glucoside (**7**) [57] which were isolated for the first time from the genus (Fig. 4). Their structures were fully elucidated using 1D and 2D NMR techniques and they were further ascertained by comparing their data with previously reported data in literature.

Dihydrodehydrodiconiferyl alcohol 4-*O*-*β*-D-glucoside (**6**) was isolated as a yellowish white amorphous powder. ^1^H-NMR of (**6**) revealed the presence of a 1,2,4-trisubstituted benzene ring with signals at *δ*_H_ 7.03 (*d*, *J* = 1.84 Hz), 7.14 (*d*, *J* = 8.43 Hz) and 6.93 (*dd*, *J* = 8.31, 2.03 Hz) for H-2, H-5 and H-6, respectively, each integrated for one proton. Also, a 1,2,3,5-tetrasubstituted benzene ring was presented by two broad singlet signals at *δ*_H_ 6.73 (H-2′) and 6.72 (H-6′). The spectrum revealed the existence of a hydroxypropyl group showing three signals at *δ*_H_ 2.63 (*t*, H-7′), 1.82 (*m*, H-8′), 3.57 (*t*, H-9′). Furthermore, a methine-methine-methylene group (CH-CH-CH_2_) appeared at *δ*_H_ 5.56 (*d*, *J* = 5.85, H-7), 3.44 (*m*, H-8), 3.80, 3.75 (*m*, *m*, H-9). The presence of *β*-D-glucose was proposed by the appearance of anomeric proton at *δ*_H_ 4.88 and other sugar protons at 3.39–3.85. Two singlet signals each integrated for three protons at *δ*_H_ 3.87 and 3.83 were attributed to two methoxy groups. ^13^C-NMR spectrum of compound (**6**) showed the presence of five aromatic methines and seven quaternary aromatic carbons signals attributed to two benzene rings at *δ*_C_ 111.19, 117.95, 119.37, 114.19, 118.04, 137.09, 150.9, 147.6, 138.37, 145.24, 147.6, 129.58. Downfield shifts of C-3 (150.9), C-4 (147.6), C-3′ (145.24), C-4′ (147.6) indicated their attachment to oxygenated functional groups. A signal at *δ*_C_ 102.78 was attributed to the anomeric carbon of glucose unit and the other sugar carbons appeared at 74.90, 77.84, 71.34, 78.19 and 62.51. The two signals at *δ*_C_ 56.79 and 56.71 represented two methoxy groups. Other aliphatic signals appeared at *δ*_C_ 88.48, 65.07, 62.22, 55.68, 35.84 and 32.89. The HMBC spectrum showed that the methoxy group at *δ*_C_ 56.79 was placed at C-3 (150.9) and the methoxy group at *δ*_C_ 56.71 was placed at C-3′ (145.24). Also, it showed a correlation between C-6, C-2 with H-7; C-6′, C-2′ with H-7′; and C-9′ with H-7′. The correlation between C-4 with H-1″ supported the presence of the sugar at C-4. From the displayed data (Table 4) and through comparison with the previously reported literature [56], compound (**6**) was identified as dihydrodehydrodiconiferyl alcohol 4-*O*-*β*-D-glucoside which was the first time to be reported in the genus.

**Table 4:** ^1^H- and ^13^C-NMR spectroscopic data for 6 and 7

	Dihydrodehydrodiconiferyl alcohol 4-*O*-*β*-D-glucoside (6)		Dihydrodehydrodiconiferyl alcohol 9-*O*-*β*-D-glucoside (7)	
	*δ*_C_	*δ*_H_ (Mult, Int), *J* in Hz	*δ*_C_	*δ*_H_ (Mult, Int), *J* in Hz
1	137.09		134.67	
2	111.19	7.03 (*d*, 1H), 1.84	110.71	7.02 (*d*, 1H), 2.01
3	150.9		149.05	
4	147.6		147.48	
5	117.95	7.14 (*d*, 1H), 8.43	116.08	6.78 (*d*, 1H), 8.09
6	119.37	6.93 (*dd*, 1H), 8.31, 2.03	119.72	6.89 (*dd*, 1H), 8.28, 2
7	88.48	5.56 (*d*, 1H), 5.85	88.98	5.62 (*d*, 1H), 6.21
8	55.68	3.44 (*m*, 1H)	53.28	3.69 (*m*, 1H)
9	65.07	3.80, 3.75 (*m*, *m*, 2H)	72.46	4.23, 3.79 (*dd*, *m*, 2H)
3-OCH_3_	56.79	3.87 (*s*, 3H)	56.44	3.85 (*s*, 3H)
1′	138.37		136.64	
2′	114.19	6.73 (*s*, 1H)	114.19	6.75 (*s*, 1H)
3′	145.24		145.21	
4′	147.6		147.48	
5′	129.58		129.56	
6′	118.04	6.72 (*s*, 1H)	118.21	6.80 (*s*, 1H)
7′	32.89	2.63 (*t*, 2H)	32.89	2.65 (*t*, 2H)
8′	35.84	1.82 (*m*, 2H)	35.82	1.84 (*m*, 2H)
9′	62.22	3.57 (*t*, 2H)	62.23	3.59 (t, 2H)
3′-OCH_3_	56.71	3.83 (*s*, 3H)	56.77	3.88 (*s*, 3H)
1″	102.78	4.88 (covered by solvent, 1H)	104.57	4.38 (*d*, 1H), 7.79
2″	74.90	3.48 (*m*, 1H)	75.18	3.25 (*m*, 1H)
3″	77.84	3.39 (*m*, 1H)	78.07	3.31 (*m*, 1H)
4″	71.34	3.39 (*m*, 1H)	71.66	3.31 (*m*, 1H)
5″	78.19	3.39 (*m*, 1H)	78.26	3.31 (*m*, 1H)
6″	62.51	3.85, 3.69 (*m*, 2H)	62.81	3.88, 3.70 (*m* 2H)

NMR data (*δ*) were measured ^1^H-NMR (400 MHz, CD_3_OH) and ^13^C-NMR data (100 MHz, CD_3_OH)

Dihydrodehydrodiconiferyl alcohol 9-*O*-*β*-D-glucoside (**7**) was isolated as a yellowish white amorphous powder. The ^1^H-NMR and ^13^C-NMR data for this compound were similar to compound (**6**) suggesting the same neolignan nucleus; the two compounds differ in the position of the glucose moiety. The HMBC spectrum revealed the correlation between C-9 with H-1″ which supported the attachment of the sugar at C-9. The downfield shift of C-9 at *δ*_C_ 72.46 also supported the attachment of the sugar at C-9 [57] (Table 4).

### In vitro assessment of the cytotoxic activity of the isolated compounds

Additionally, the cytotoxic activity of the compounds obtained from the *n*-hexane and dichloromethane fractions of *B. rupestris* was examined using different concentrations (20, 10, 5, 2.5 μg/mL) of these compounds on the same cell lines utilized in the determination of the cytotoxic effect of the total extracts and subsequent fractions. The isolated compounds showed no cytotoxicity against hepatoma HepG2, breast MDA-MB-231 and lung A549 cancer cell lines with growth inhibition below 20%. The results are illustrated in Table 5. Doxorubicin was employed as a positive control and exhibited a strong cytotoxic effect against HepG2 (IC_50_ 0.49 μg/mL), MDA-MB-231 (IC_50_ 0.68 μg/mL) and A549 (IC_50_ 0.13 μg/mL) cells.

**Table 5:** Cytotoxic activity of the isolated compounds

Cell line	Conc. (μg/mL)	% Inhibition	
		*β*-amyrin acetate (1)	Scopoletin (4)
HepG2	20	12.1 ± 3.4	11.4 ± 2.1
	10	16.0 ± 0.3	6.5 ± 1.2
	5	20.4 ± 2.3	0.9 ± 0.1
	2.5	8.23 ± 0.7	5.9 ± 1.2
MDA-MB-231	20	−19.6 ± 1.2	8.2 ± 1.0
	10	−13.9 ± 1.2	6.0 ± 0.3
	5	−5.5 ± 1.2	10.7 ± 9.8
	2.5	7.0 ± 0.1	15.5 ± 0.3
A549	20	2.2 ± 0.5	7.9 ± 0.4
	10	9.0 ± 1.0	14.9 ± 0.4
	5	10.8 ± 0.5	14.6 ± 0.5
	2.5	0.4 ± 0.7	14.3 ± 0.7

Results are presented as cell growth inhibition percentage at concentrations of 2.5 to 20 μg/mL, mean ± SD (*n* = 3). Doxorubicin was used as the positive control and exerted significant cell viability inhibitory effects against HepG2 (IC_50_ 0.49 μg/mL), MDA-MB-231 (IC_50_ 0.68 μg/mL)

### In vitro assessment of the anti-allergic activity of the isolated compounds

To ascertain, whether the isolated compounds might be responsible for the anti-allergic activity observed in *Brachychiton* sp. crude extracts and nonpolar fractions, the isolated compounds were subjected to degranulation assay in RBL-2H3 mast cell model. The results are presented in Table 6. MTT viability assay was used to evaluate the potential toxic effects against RBL-2H3 cells. A mixture of *β*-sitosterol (**2**) and stigmasterol (**3**) (200 and 100 μg/mL) was considered toxic (viability below 85%). According to our results, scopoletin (**4**) showed 23.0% inhibition of A23187-induced and 30.0% of antigen-induced degranulation at 500 μM. Dihydrodehydrodiconiferyl alcohol 9-*O*-*β*-D-glucoside (**7**) showed only weak inhibitory effect in the A23187-induced assay (16.3% at 100 μM and 18.0% at 500 μM). Dexamethasone (10 nM) was utilized as the positive control and inhibited *β*-hexosaminidase release by 93.7%.

**Table 6:** Anti-allergic activity of compounds isolated from *B. rupestris*

Sample	% viability, RBL-2H3^a^		% inhibition of A23187-induced *β*-hexosaminidase release^b^		
	100 μM	500 μM	10 μM	100 μM	500 μM
*β*-amyrin acetate (1)	98.3 ± 2.9	^c^	0.3 ± 0.6	0.7 ± 1.2	^c^
scopoletin (4)	96.0 ± 4.0	93.7 ± 6.5	3.7 ± 4.7	13.7 ± 8.0	23.0 ± 8.0^**d^
*β*-sitosterol-3-O-*β*-D-glucoside (5)	96.0 ± 1.0	^c^	5.0 ± 5.6	7.7 ± 3.8	^c^
dihydrodehydrodiconiferyl alcohol 4-*O*-*β*-D-glucoside (6)	97.3 ± 2.5	^e^	7.0 ± 10.4	12.7 ± 7.6	^e^
dihydrodehydrodiconiferyl alcohol 9-*O*-*β*-D-glucoside (7)	97.7 ± 2.1	95.3 ± 4.2	3.3 ± 4.2	16.3 ± 5.5*	18.0 ± 8.7^*^

A mixture of *β*-sitosterol (2) and stigmasterol (3) was toxic towards RBL-2H3 cells at the concentration of 200 μg/mL (73.7 ± 11.7% viability) and 100 μg/ml (78.7 ± 11.6% viability) and inactive at the concentration of 10 μg/mL (5.0% ± 5.0% inhibition) in A23187-induced degranulation assay

^a^The cytotoxicity of samples to RBL-2H3 was evaluated using MTT viability assay; results are presented as mean ± SD (*n* = 3)

^b^Dexamethasone (10 nM) was used as the positive control and inhibited 93.7 ± 1.5%^**^ of A23187-induced *β*-hexosaminidase release in RBL-2H3 cells. Results are presented as mean ± SD (*n* = 3); ^*^p < 0.05, ^**^p < 0.001 compared with the control value (A23187 only)

^c^Precipitate was formed upon the addition into the medium at the concentration of 500 μM, therefore the result could not be justified

^d^Scopoletin (500 μM) exerted 30.0 ± 7.1% inhibition of antigen-induced *β*-hexosaminidase release (mean ± SD, *n* = 2)

^e^Dihydrodehydrodiconiferyl alcohol 4-*O*-*β*-D-glucoside was not tested at the concentration of 500 *μ*M, however, it was nontoxic towards RBL-2H3 cells (96.0 ± 6.9% viability) and inactive in A23187-induced degranulation assay (10.0 ± 4.6% inhibition) at the concentration of 200 *μ*M

### In vitro assessment of the anti-inflammatory activity of the isolated compounds

The anti-inflammatory effect of the isolated compounds was determined to understand whether any of these compounds might be accountable for the potent activity of *B. rupestris* crude extract and its fractions. The results are illustrated in Table 7. According to the results, scopoletin (**4**) was found to significantly inhibit elastase release in fMLF-induced human neutrophils by 22.8% at 10 μM. Genistein, natural tyrosine kinase inhibitor [50, 51], was used as the positive control and caused significant suppression of superoxide anion generation (IC_50_ 1.16 μM) and elastase release (IC_50_ 21.51 μM).

**Table 7:** Effect of pure compounds on superoxide anion generation and elastase release in fMLF/CB-induced human neutrophils

Sample	Superoxide anion generation^a^	Elastase release^a^
	IC_50_ (μΜ)^b^	IC_50_ (μΜ)^b^
*β*-amyrin acetate (1)	> 10	> 10
scopoletin (4)	> 10	> 10^c^
*β*-sitosterol-3-*O*-*β*-D-glucoside (5)	> 10	> 10
dihydrodehydrodiconiferyl alcohol 4-*O*-*β*-D-glucoside (6)	> 10	> 10
dihydrodehydrodiconiferyl alcohol 9-*O*-*β*-D-glucoside (7)	>1^d^	>1^d^
genistein	1.16 ± 0.12	21.51 ± 6.50

^a^IC_50_ values, results are presented as mean ± SD (*n* = 3–4), compared with the control value (formyl-methionyl-leucyl-phenylalanine/cytochalasin B, fMLF/CB)

^b^Concentration necessary for 50% inhibition (IC_50_)

^c^Scopoletin (4) exerted significant inhibitory activity in elastase release assay (22.8 ± 15.3%, *p < 0.05) at 10 μM

^d^Dihydrodehydrodiconiferyl alcohol 9-*O*-*β*-D-glucoside (10) was used at the final concentration of 1 μM due to solubility issues

## Discussion

RBL-2H3 are mast cells that greatly affect the development of allergic response [58]. Upon activation by antigen or A23187 (calcium ionophore), mast cells produce histamine in addition to other mediators that immediately initiate hypersensitivity reactions. *β*-Hexosaminidase represents an important mast cells degranulation marker that is commonly used for the assessment of anti-allergic activity [59].

The anti-allergic activity of the crude extracts as well as *n*-hexane and dichloromethane fractions of *B. rupestris* and *B. discolor* leaves (Table 2) might be attributed to the presence of many active constituents from their non-polar fractions. Sterols, sterol glycosides, coumarin, and triterpenes were isolated and identified in *B. rupestris* leaves*.* Lanosterol, lupeol, *β*-amyrin, *β*-amyrin acetate and oleanolic acid were previously reported from *B. discolor* leaves by Kassem et al. [23]. These triterpenes were reported to exert a potent anti-allergic activity [60–62] including *β*-amyrin that was previously documented to exhibit mast cell membrane stabilization [30]. The anti-allergic activity of triterpenes might be attributed to the suppression of secretion of histamine and interleukins (IL-2, IL-4) from mast cells [62]. Also, *β*-sitosterol was reported to possess anti-allergic activity and might have therapeutic potential in allergic asthma [63, 64]. It was suggested that *β*-sitosterol and its glycoside inhibited the release of IL-4 so it could act as an immune modulator to relieve symptoms associated with seasonal allergic response [65]. However, we did not observe any significant effect of either *β*-amyrin acetate (**1**), the mixture of *β*-sitosterol (**2**) and stigmasterol (**3**) or *β*-sitosterol glycoside (**5**) in degranulation assay using the RBL-2H3 mast cell model (Table 6). Meanwhile, scopoletin (**4**) and dihydrodehydrodiconiferyl alcohol 9-*O*-*β*-D-glucoside (**7**) showed inhibitory activity on degranulation in RBL-2H3 cells.

Regarding the in vitro anti-inflammatory activity, neutrophils exert a vital role in host’s defenses versus the attack by microorganisms and in the pathogenesis of various inflammatory diseases [66]. In response to stimuli, such as fMLF, the activated neutrophils secrete a series of inflammatory mediators such as superoxide anion (O_2_^.−^) and elastase which are major contributors to the destruction of tissue in inflammatory response [67]. We observed that the crude extracts and fractions of *B. rupestris* and *B. discolor* leaves (Table 3) exerted potent anti-inflammatory activity in human neutrophils. Many studies supported the anti-inflammatory activity of sterols and their glycosides [68–70]. They induce immunomodulatory response that affects inflammatory mediators [71, 72]. They were also reported to possess potent in vivo anti-inflammatory activity with the concomitant reduction of edema and inflammation in rats [73]. Various studies confirmed the anti-inflammatory activity of triterpenes including *β-amyrin and β-amyrin acetate* [29, 74–76]. Coumarins also were found to have anti-inflammatory activity by inhibiting different inflammatory mediators such as cyclooxygenase-2, nitric oxide, tumor necrosis factor-*α* and interleukins [77–79]. Neolignans were reported to exert anti-inflammatory effects by suppressing superoxide anion generation and elastase release [80], they also exhibited nitric oxide (NO) and tumor necrosis factor-α (TNF-α) inhibitory effects [81]. However, according to our results, only scopoletin (**4**) exerted mild inhibition of elastase release. All other isolated compounds including dihydrodehydrodiconiferyl alcohol glycosides (**6** and **7**) were inactive in fMLF-activated human neutrophils.

## Conclusions

The total extract and fractions of *B. rupestris* and *B. discolor* were nontoxic against hepatoma, breast and lung cancer cell lines. The crude extracts as well as the *n*-hexane and dichloromethane fractions of *B. rupestris* and *B. discolor* exhibited significant anti-allergic as well as anti-inflammatory activities. The phytochemical study of the leaves of *B. rupestris* resulted in the isolation of compounds from different chemical classes, including triterpene, sterols, sterol glycoside, coumarin and neolignans. All the tested compounds were nontoxic against the tested cancer cell lines. Among the isolated compounds, scopoletin exerted anti-allergic effects and mild anti-inflammatory activity by reducing elastase release in human neutrophils. However, the bioactivity of *B. rupestris* extracts and fractions was much more potent compared with any of the isolated compounds. Thus, leaves of *B. rupestris* and *B. discolor* are worth to be considered for further development and research based on their anti-allergic and anti-inflammatory activities. In vivo evaluation of the anti-allergic and anti-inflammatory activities is highly recommended for the active fractions of both *Brachychiton* species.

## Additional file

Additional file 1: Supplementary data contains supplementary figures (Figures S1-S6) showing the spectroscopic data of isolated compounds 1–**7** from *n*-hexane, dichloromethane and ethyl acetate fractions of *B. rupestris*. (DOCX 2306 kb)

## Abbreviations

ANOVA: One-way analysis of variance
APT: Attached proton test
*B*: *Brachychiton*
BHT: tert-Butyl-1-hydroxytoluene
BDD: *Brachychiton discolor* leaves dichloromethane fraction
BDE: *Brachychiton discolor* leaves ethyl acetate fraction
BDH: *Brachychiton discolor* leaves *n*-hexane fraction
BDT: *Brachychiton discolor* leaves total extract
BRD: *Brachychiton rupestris* leaves dichloromethane fraction
BRE: *Brachychiton rupestris* leaves ethyl acetate fraction
BRH: *Brachychiton rupestris* leaves *n*-hexane fraction
BRR: *B. rupestris* remaining fraction
BRT: *Brachychiton rupestris* leaves total extract
CDCL_3_: Deuterated chloroform
CD_3_OD: Deuterated methanol
^13^C-NMR: Carbon-13 Nuclear Magnetic Resonance
*d*: doublet
*dd*: doublet of doublet
DMEM: Dulbecco’s modified Eagle’s medium
DMSO: Dimethylsulfoxide
DMSO-d6: Deuterated dimethylsulfoxide-d6
2D-NMR: Two-dimensional nuclear magnetic resonance spectroscopy
DNP: Dinitrophenyl
DNP-BSA: Dinitrophenyl-conjugated bovine serum albumin
ESI-MS: Electro-Spray Ionization Mass Spectrometry
EtOAc: Ethyl acetate
FBS: Fetal bovine serum
fMLF/CB: formyl-methionyl-leucyl-phenylalanine/cytochalasin B
^1^H-^1^H COSY: ^1^H-^1^H Correlated spectroscopy
HMBC: Heteronuclear Multiple-Bond Correlation Spectroscopy
^1^H-NMR: Proton Nuclear Magnetic Resonance
HSQC: Heteronuclear Single Quantum Coherence
IC_50_: The half maximal inhibitory concentration
*m*: multiplet
MTT: (3-(4,5-dimethylthiazol-2-yl)-2,5-diphenyltetrazolium bromide)
*p*-NAG: *p*-Nitrophenyl-N-acetyl-D-glucosaminide
PTLC: Preparative thin layer chromatography
*q*: quartet
R_*f*_: Retardation factor
*s*: Singlet
S.D.: Standard deviation
SEM: Standard error of mean
*t*: Triplet
TLC: Thin Layer Chromatography
UPLC: Ultra Performance Liquid Chromatography
