# Supplementary material for: Study of the anti-allergic and anti-inflammatory activity of Brachychiton rupestris and Brachychiton discolor leaves (Malvaceae) using in vitro models
Source: BMC Complement Altern Med. 2018 Nov 9;18:299. doi: 10.1186/s12906-018-2359-6 (PMC6230296; doi:10.1186/s12906-018-2359-6)
Supplement: Supplementary file 1 — Supplementary data contains supplementary figures (Figures S1-S6) showing the spectroscopic data of isolated compounds 1–7 from n-hexane, dichloromethane and ethyl acetate fractions of B. rupestris. (DOCX 2306 kb) [file 12906_2018_2359_MOESM1_ESM.docx]

**Study of the anti-allergic and anti-inflammatory activity of *Brachychiton rupestris* and *Brachychiton discolor* leaves (Malvaceae) using *in vitro* models**

Amany A. Thabet^1^**^†^**, Fadia S. Youssef^1^**^†^**, Michal Korinek^2,3,4,5^, Fang-Rong Chang^2,6^, Yang-Chang Wu^2,7,8^, Bing-Hung Chen^3,8,9^, Mohamed El-Shazly^1,10*^ and Abdel Nasser B. Singab^1*^ and Tsong-Long Hwang^4,5,11,12,13*^

^1^Department of Pharmacognosy, Faculty of Pharmacy, Ain Shams University, African Union Organization Street, Abbassia 11566, Cairo, Egypt

^2^Graduate Institute of Natural Products, College of Pharmacy, Kaohsiung Medical University, Kaohsiung 80708, Taiwan

^3^Department of Biotechnology, College of Life Science, Kaohsiung Medical University, Kaohsiung 80708, Taiwan

^4^Graduate Institute of Natural Products, College of Medicine, Chang Gung University, Taoyuan 33302, Taiwan

^5^Research Center for Chinese Herbal Medicine, Research Center for Food and Cosmetic Safety, and Graduate Institute of Health Industry Technology, College of Human Ecology, Chang Gung University of Science and Technology, Taoyuan 33302, Taiwan

^6^National Research Institute of Chinese Medicine, Ministry of Health and Welfare, Taipei 11221, Taiwan

^7^Research Center for Natural Products & Drug Development, Kaohsiung Medical University, Kaohsiung 80708, Taiwan

^8^Department of Medical Research, Kaohsiung Medical University Hospital, Kaohsiung 80708, Taiwan

^9^The Institute of Biomedical Sciences, National Sun Yat-sen University, Kaohsiung 80424, Taiwan

^10^Department of Pharmaceutical Biology, Faculty of Pharmacy and Biotechnology, German University in Cairo, Cairo 11835, Egypt

^11^Department of Anesthesiology, Chang Gung Memorial Hospital, Taoyuan 33305, Taiwan

^12^Chinese Herbal Medicine Research Team, Healthy Aging Research Center, Chang Gung University, Taoyuan 33302, Taiwan

^13^Department of Chemical Engineering, Ming Chi University of Technology, New Taipei City 24301, Taiwan

**^†^**These authors have contributed equally to this work

***Corresponding authors:**

Assoc. Professor Mohamed El-Shazly; Tel: +201-001401091; Fax: +202-24051107; E-mail:

[mohamed.elshazly@pharma.asu.edu.eg](mailto:mohamed.elshazly@pharma.asu.edu.eg) (M. El-Shazly). Department of Pharmacognosy, Faculty of Pharmacy, Ain Shams University, African Union Organization Street, Abbassia 11566, Cairo, Egypt.

Professor Dr. Abdel Nasser B. Singab; Tel: +201-005036231; Fax: +202-24051107; E-mail: [dean@pharma.asu.edu.eg](mailto:dean@pharma.asu.edu.eg) (B. Singab); Department of Pharmacognosy, Faculty of Pharmacy, Ain Shams University, African Union Organization Street, Abbassia 11566, Cairo, Egypt.

Professor Tsong-Long Hwang, Tel: +886-3-2118800 ext. 5523; Email: [htl@mail.cgu.edu.tw](mailto:htl@mail.cgu.edu.tw) (T.-L. Hwang); Graduate Institute of Natural Products, College of Medicine, Chang Gung University, Taoyuan 33302, Taiwan

**Supporting information**


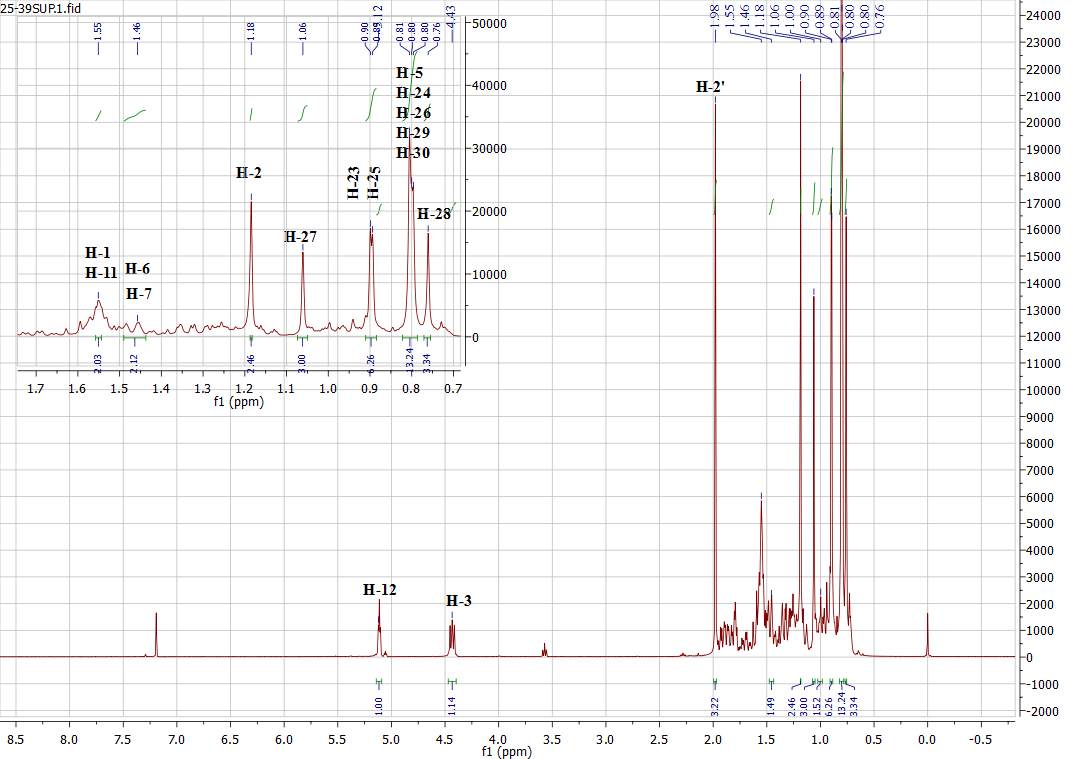


**Fig. S1a**: ^1^H NMR spectrum of *β*-amyrin acetate (**1**)


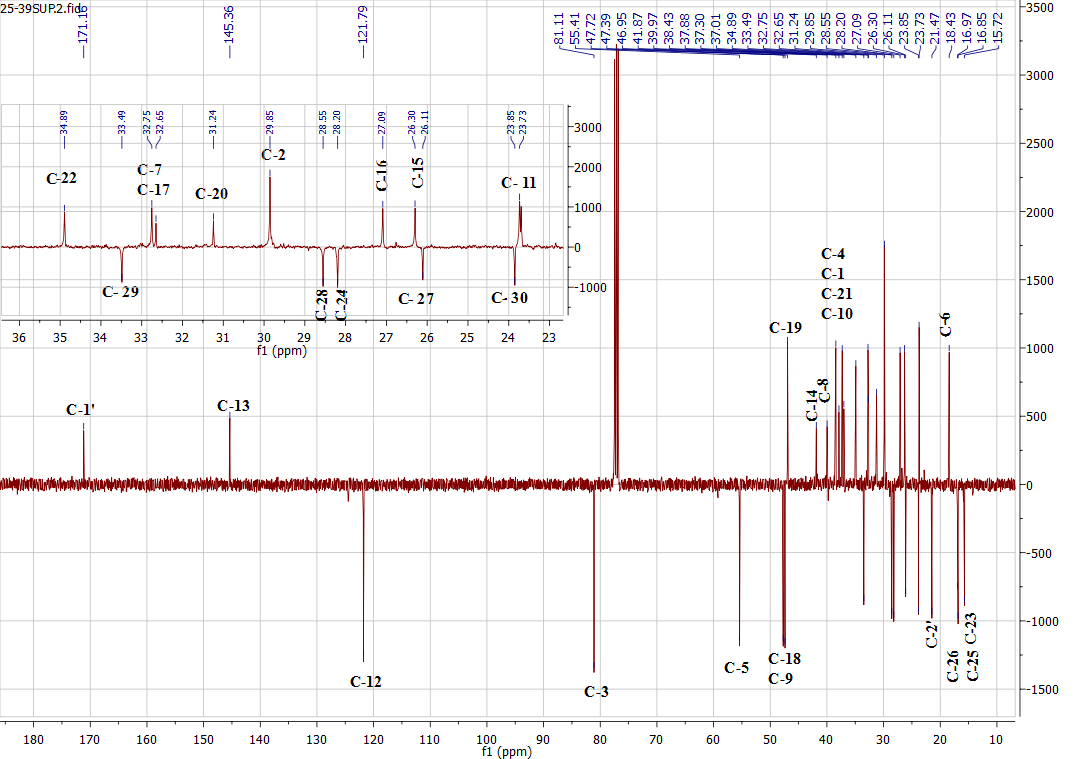


**Fig. S1b**: APT spectrum of *β*-amyrin acetate (**1**)


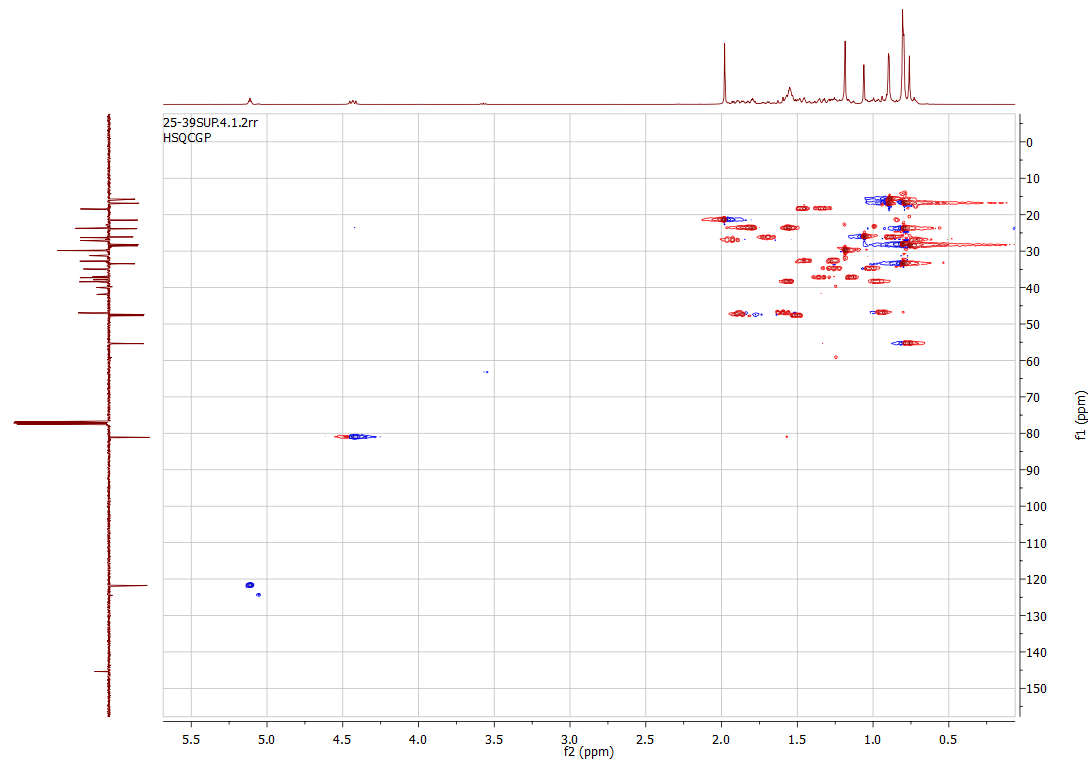


**Fig. S1c**: HSQC spectrum of *β*-amyrin acetate (**1**)


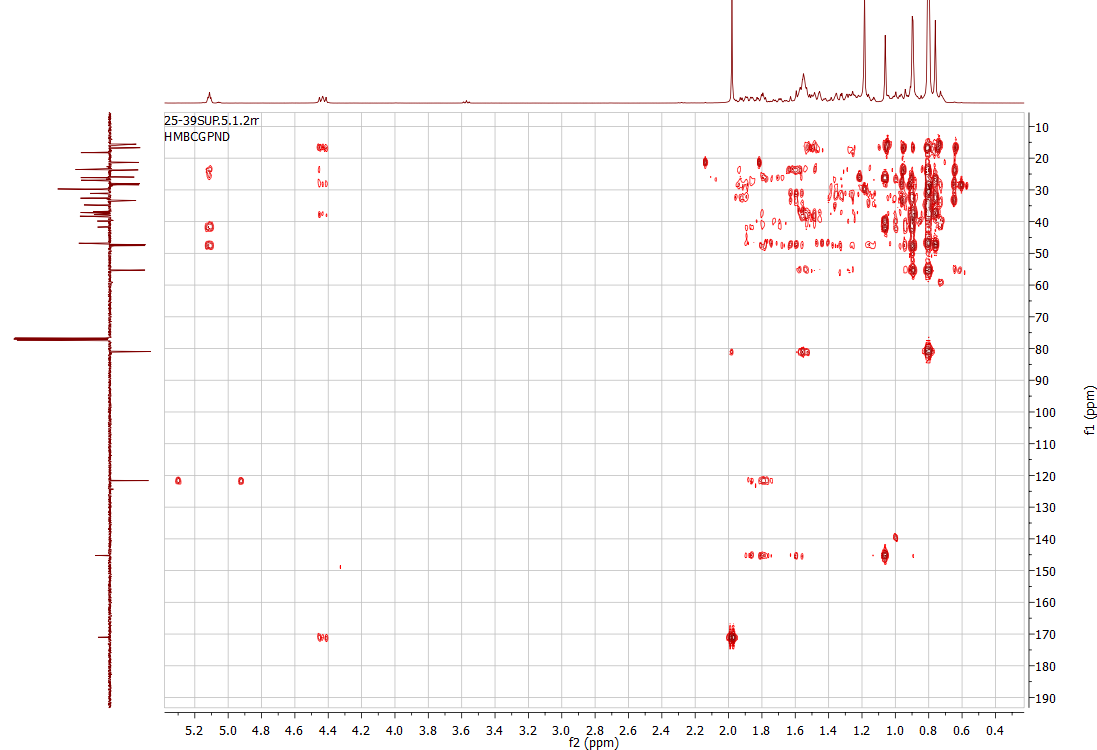


**Fig. S1d**: HMBC spectrum of *β*-amyrin acetate (**1**)


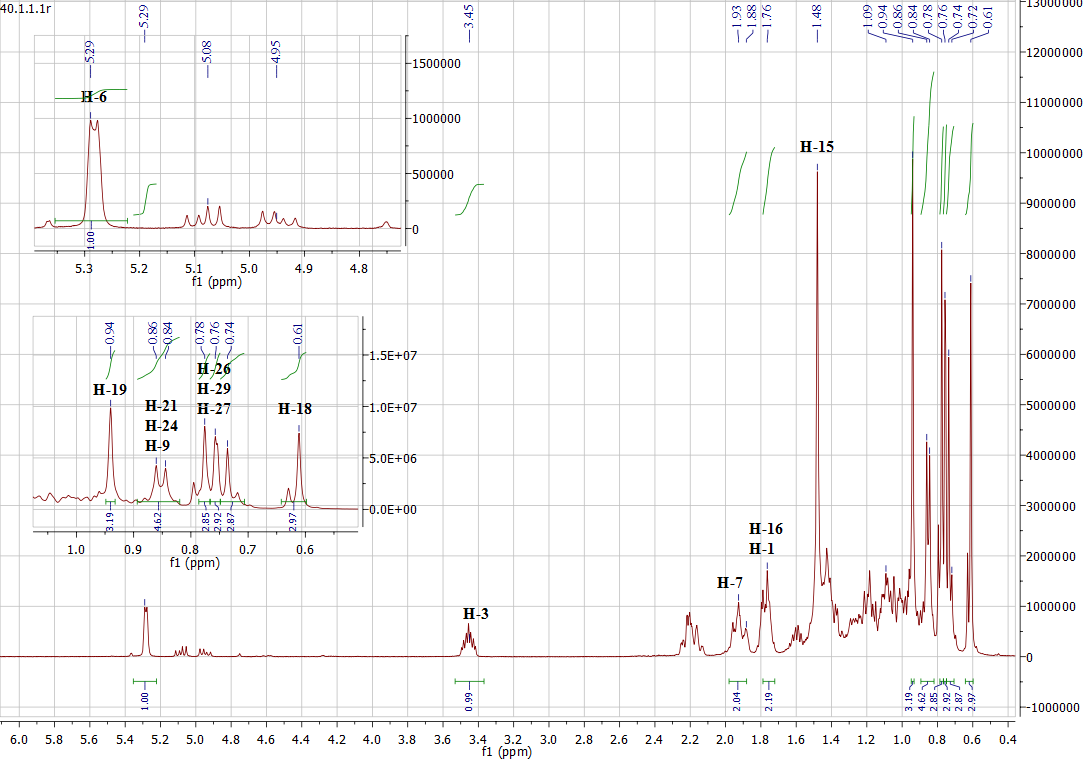


**Fig. S2a:** ^1^H NMR spectrum of *β*-sitosterol (**2**) and stigmasterol (**3**)


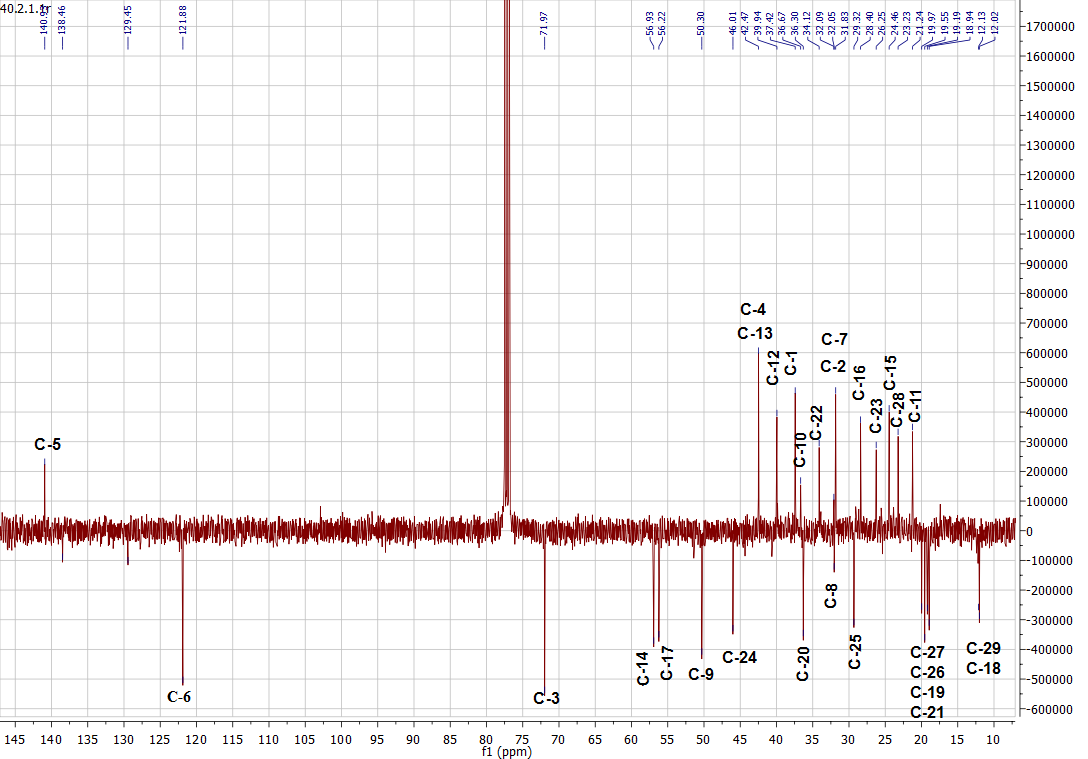


**Fig. S2b:** APT spectrum of *β*-sitosterol (**2**) and stigmasterol (**3**)


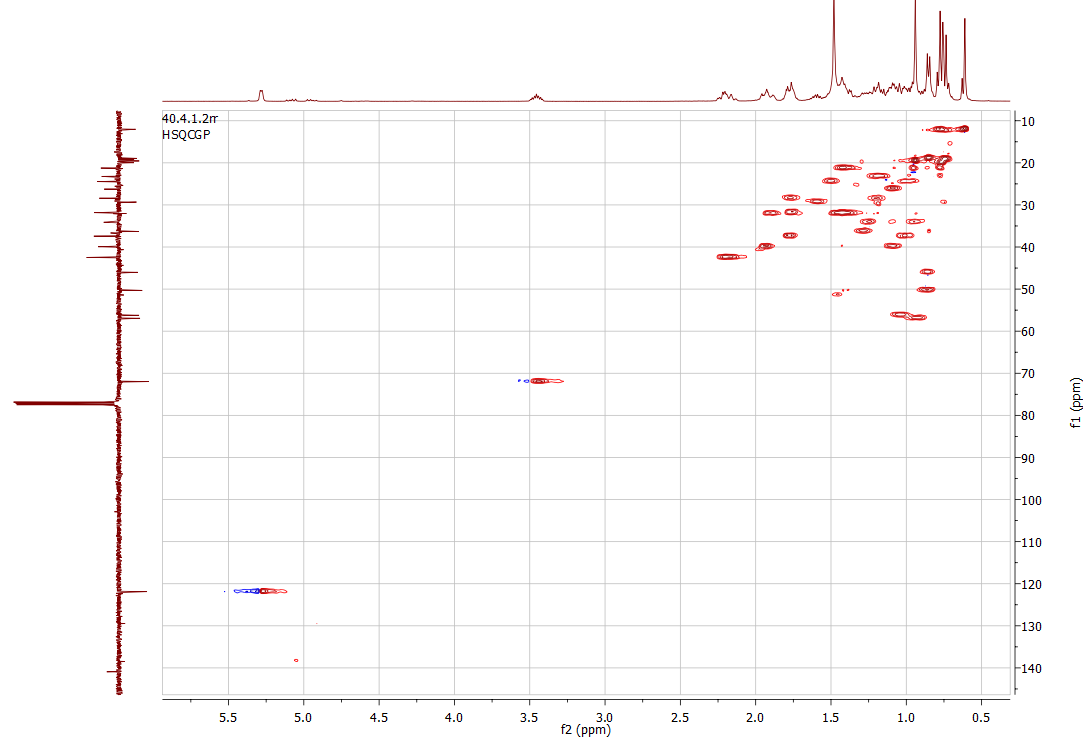


**Fig. S2c:** HSQC spectrum of *β*-sitosterol (**2**) and stigmasterol (**3**)


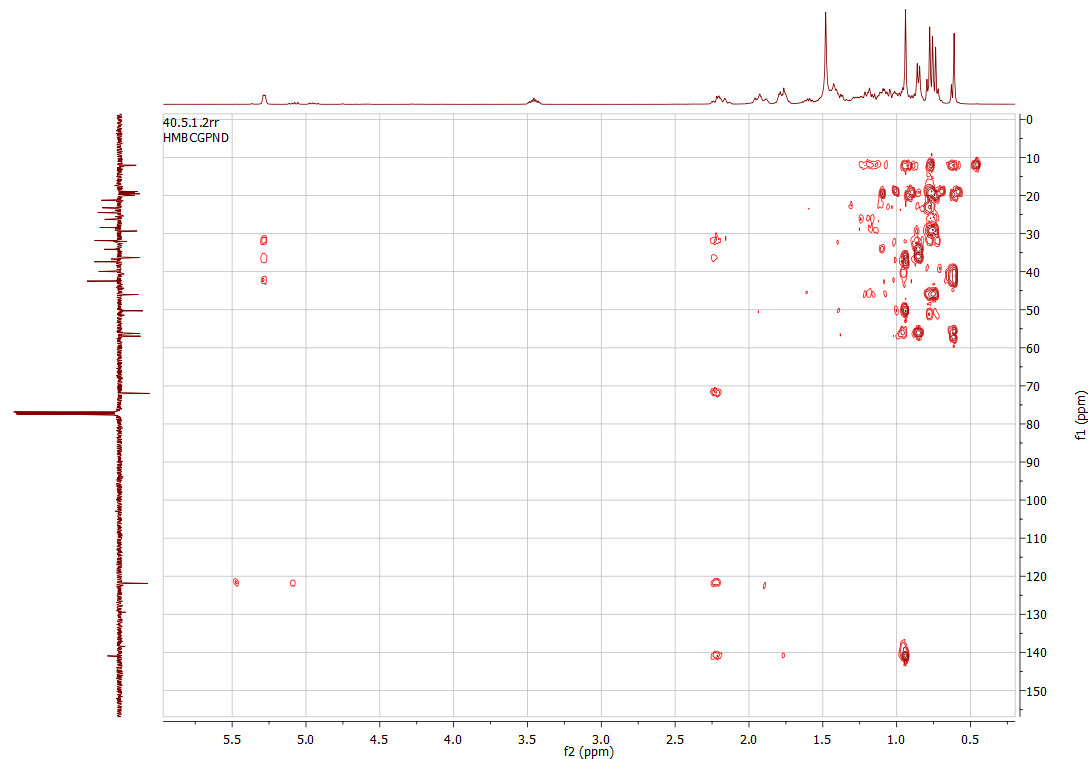


**Fig. S2d:** HMBC spectrum of *β*-sitosterol (**2**) and stigmasterol (**3**)


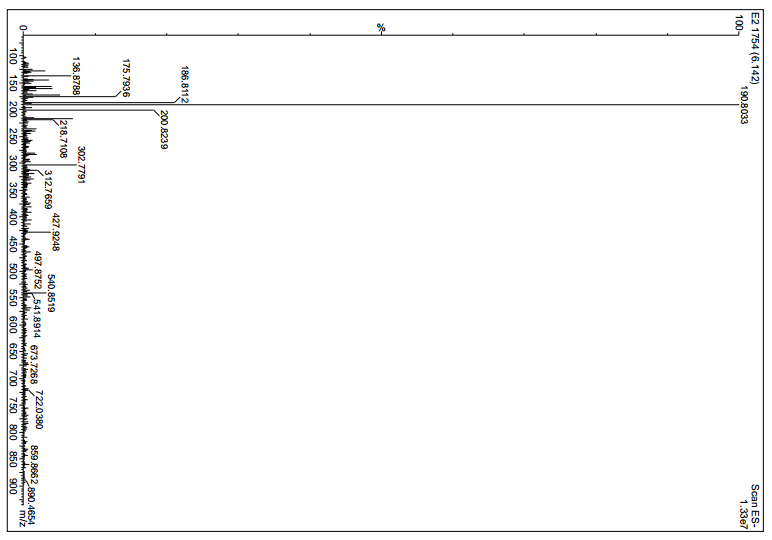


**Fig. S3a:** ESI^-^-MS spectrum of scopoletin (**4**)

**[M – H]^+^**


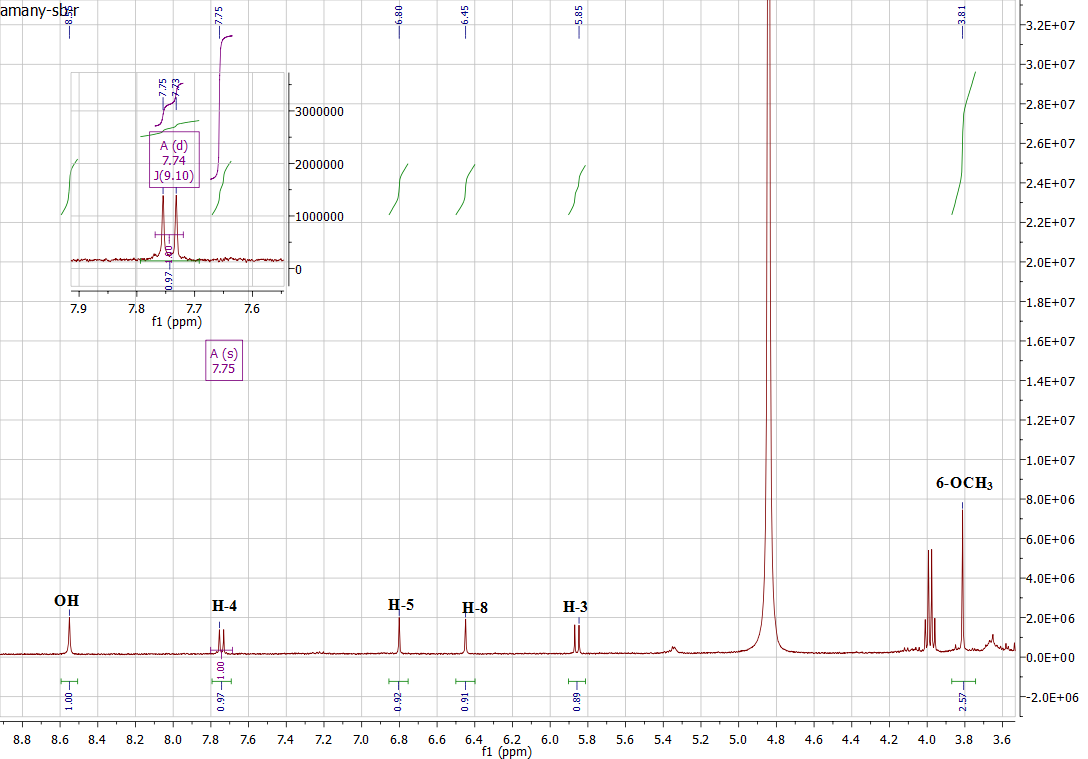


**Fig. S3b:** ^1^H NMR spectrum of scopoletin (**4**)


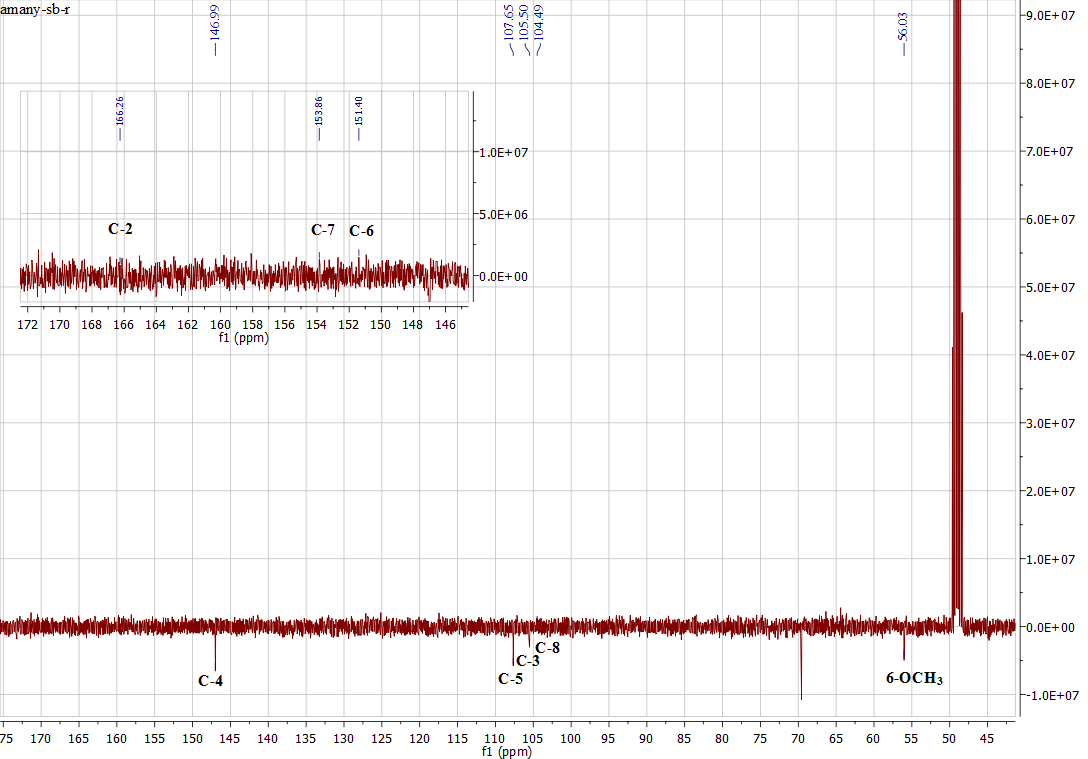


**Fig. S3c:** APT spectrum of scopoletin (**4**)


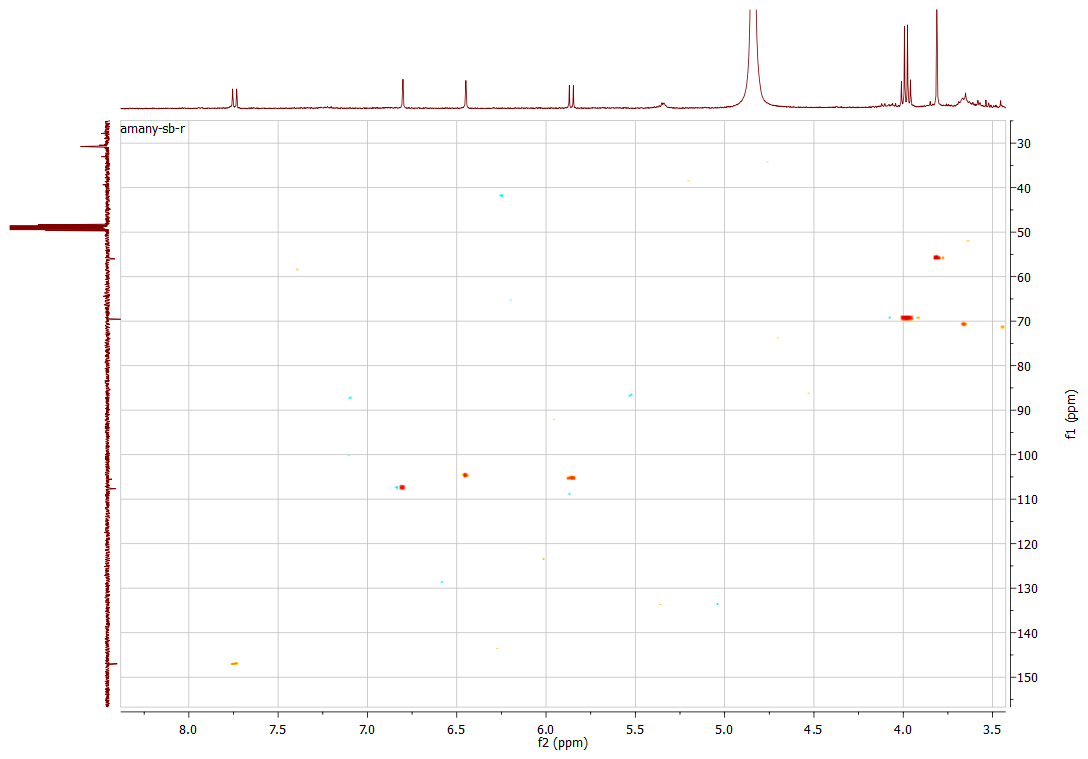


**Fig. S3d:** HSQC spectrum of scopoletin (**4**)


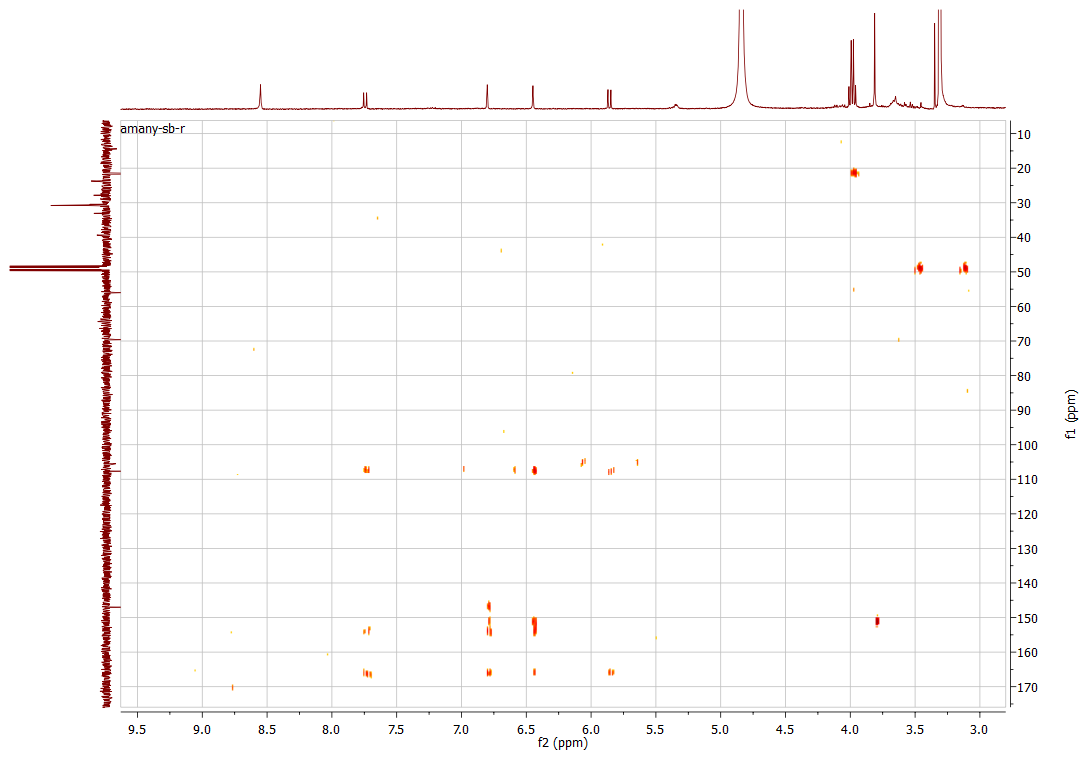


**Fig. S3e:** HMBC spectrum of scopoletin (**4**)


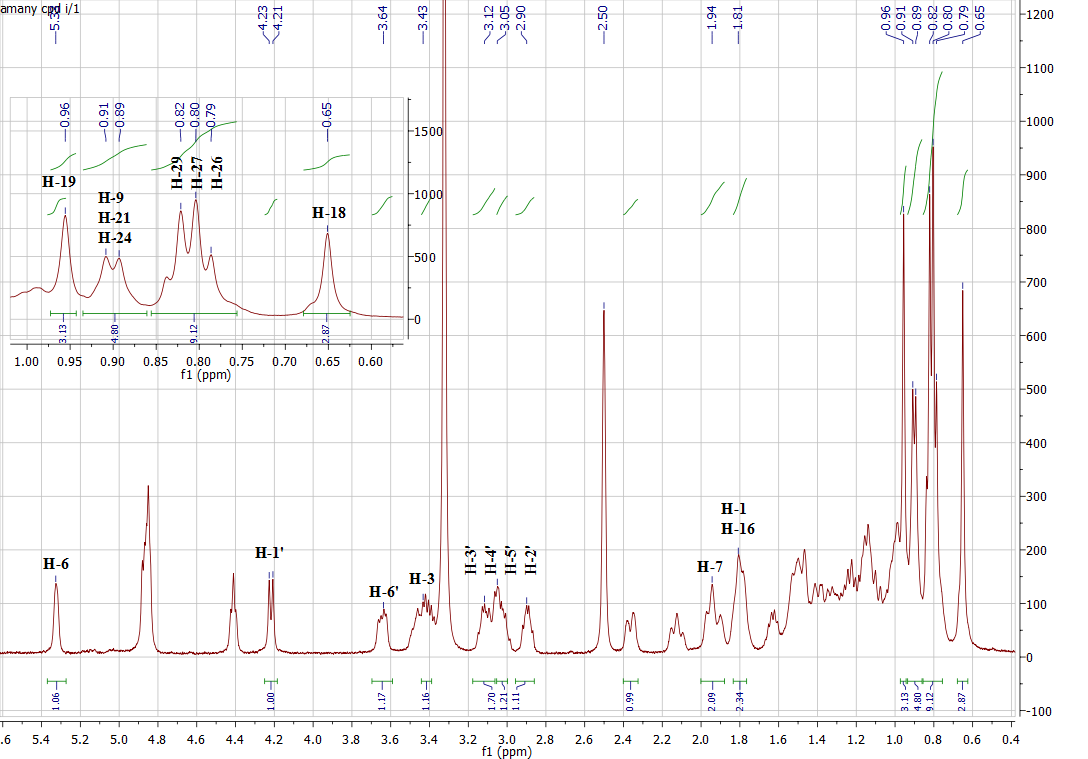


**Fig. S4a:** ^1^H NMR spectrum of *β*-sitosterol-3-*O*-*β*-D-glucoside (**5**)

**
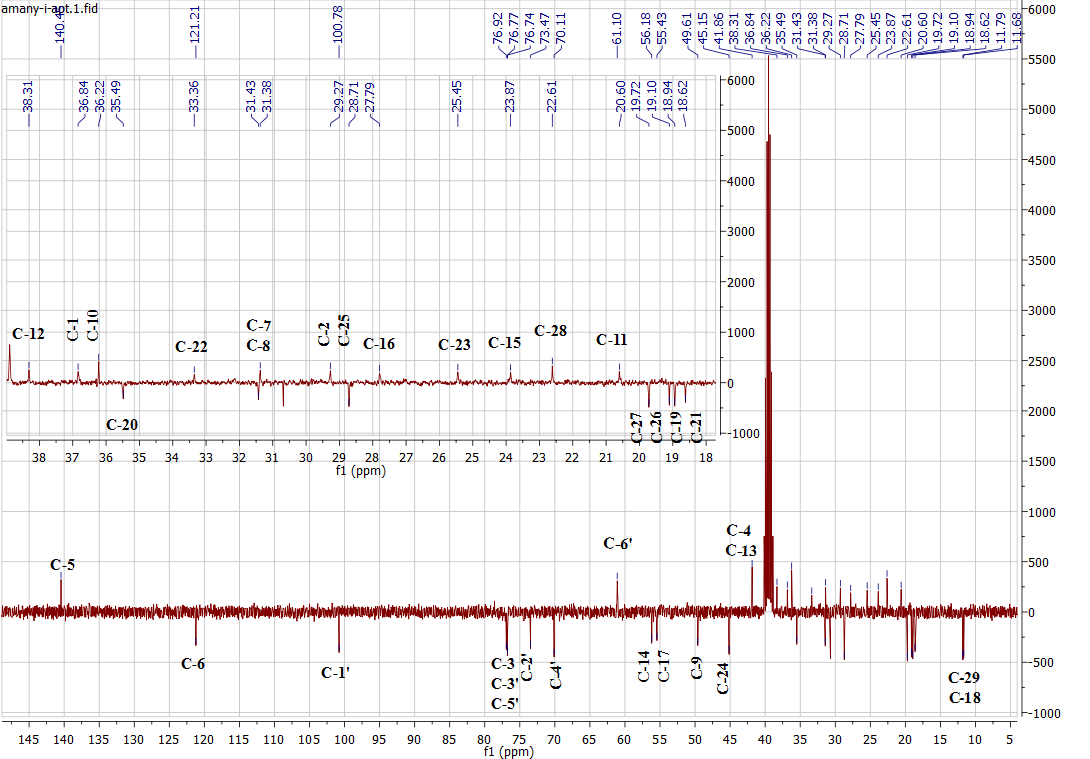
**

**Fig. S4b:** APT spectrum of *β*-sitosterol-3-*O*-*β*-D-glucoside (**5**)

**
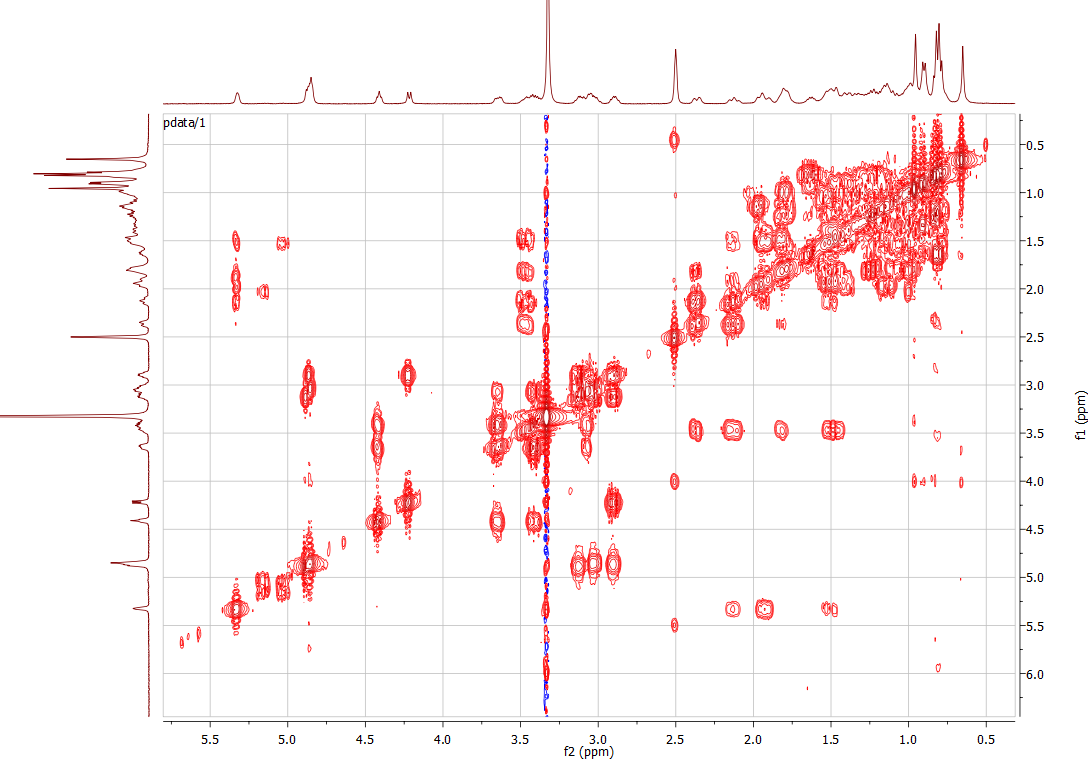
**

**Fig. S4c:** ^1^H,^1^H COSY spectrum of *β*-sitosterol-3-*O*-*β*-D-glucoside (**5**)

**
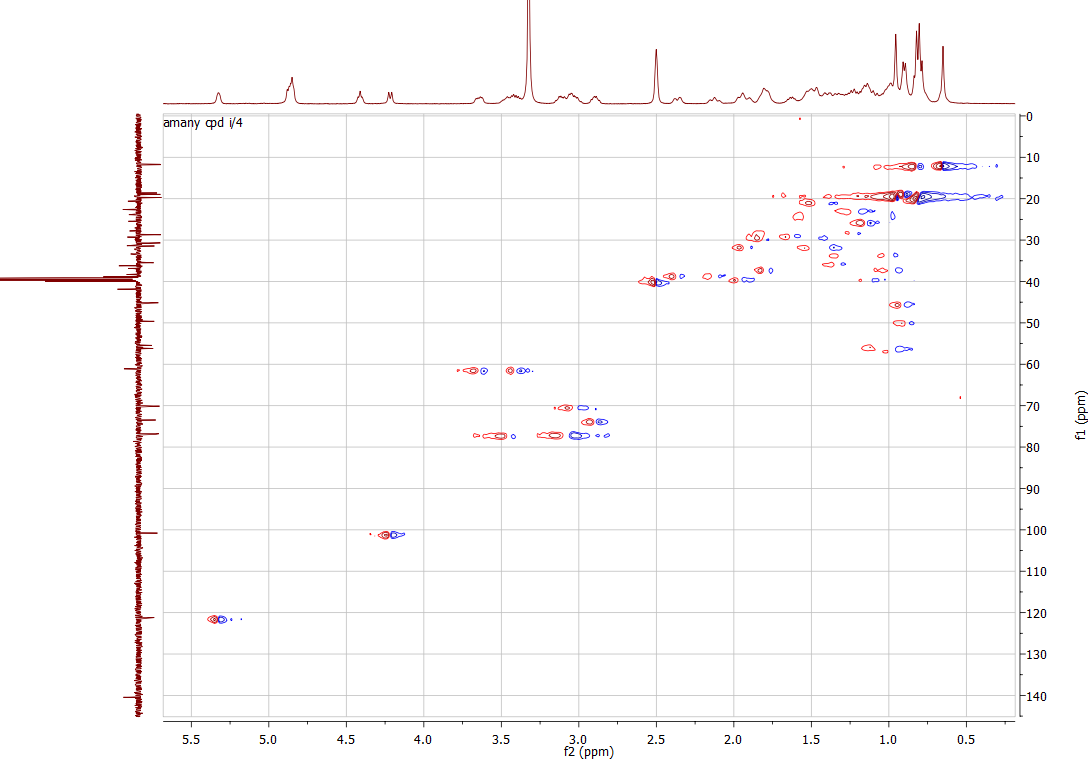
**

**Fig. S4d:** HSQC spectrum of *β*-sitosterol-3-*O*-*β*-D-glucoside (**5**)

**
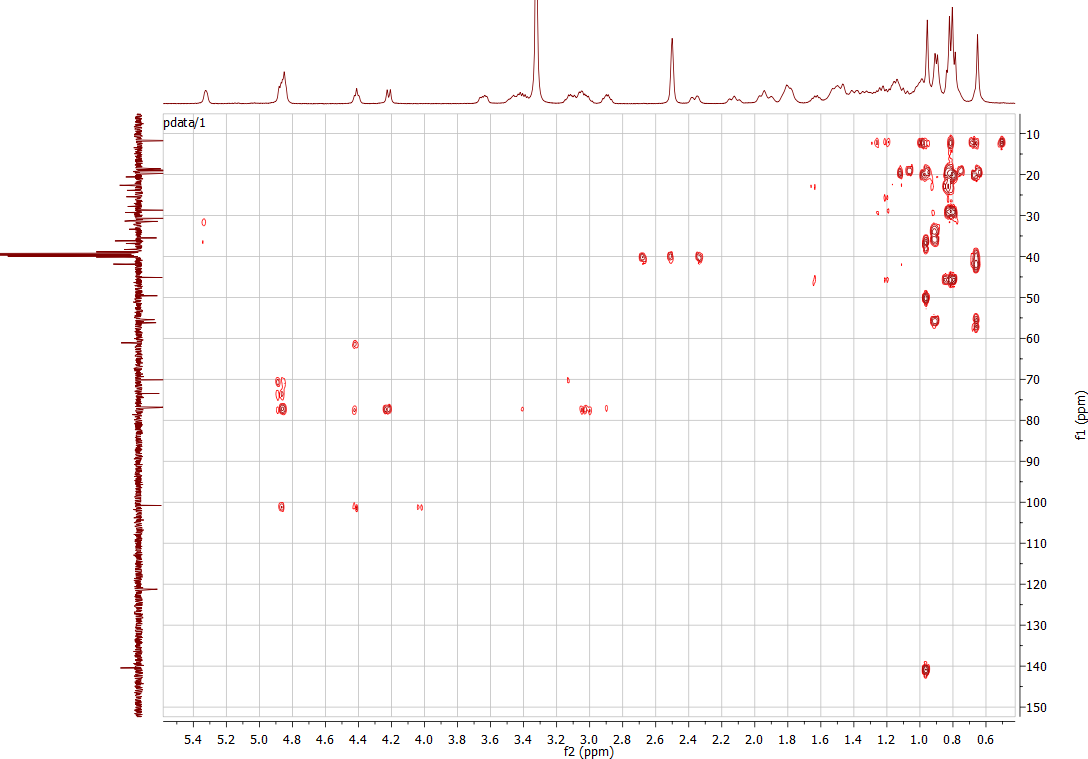
**

**Fig. S4e:** HMBC spectrum of *β*-sitosterol-3-*O*-*β*-D-glucoside (**5**)


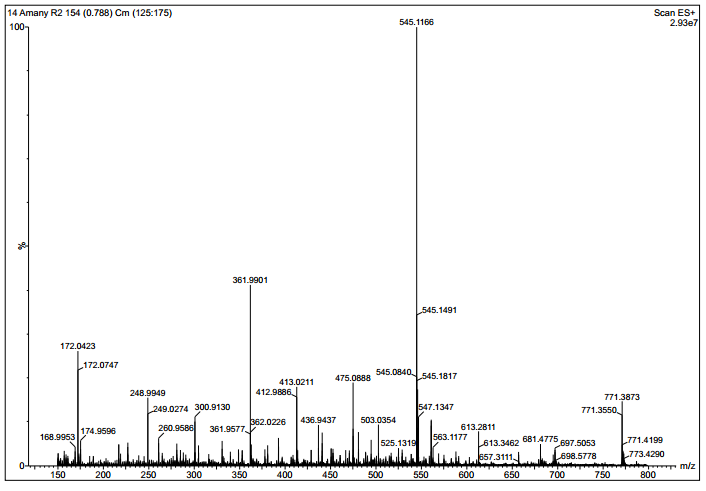


**[M + Na]^+^**

**Fig. S5a:** ESI^+^-MS spectrum of dihydrodehydrodiconiferyl alcohol 4-*O*-*β*-D-glucoside (**6**)

**
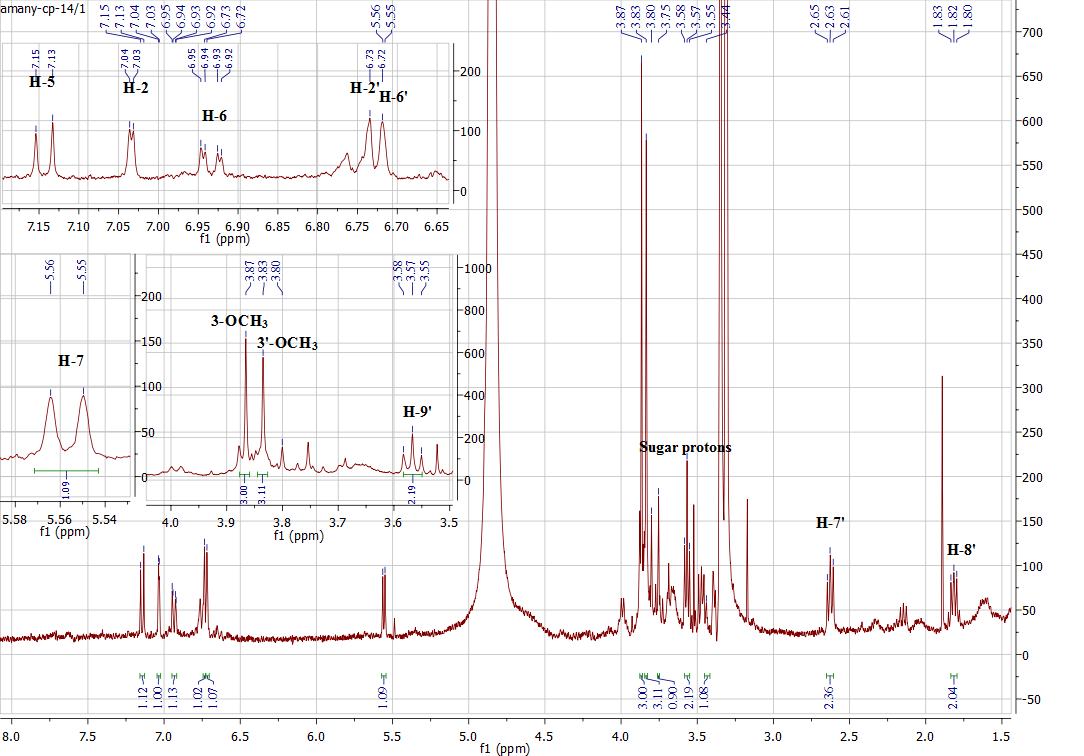
**

**Fig. S5b:** ^1^H NMR spectrum of dihydrodehydrodiconiferyl alcohol 4-*O*-*β*-D-glucoside (**6**)

**
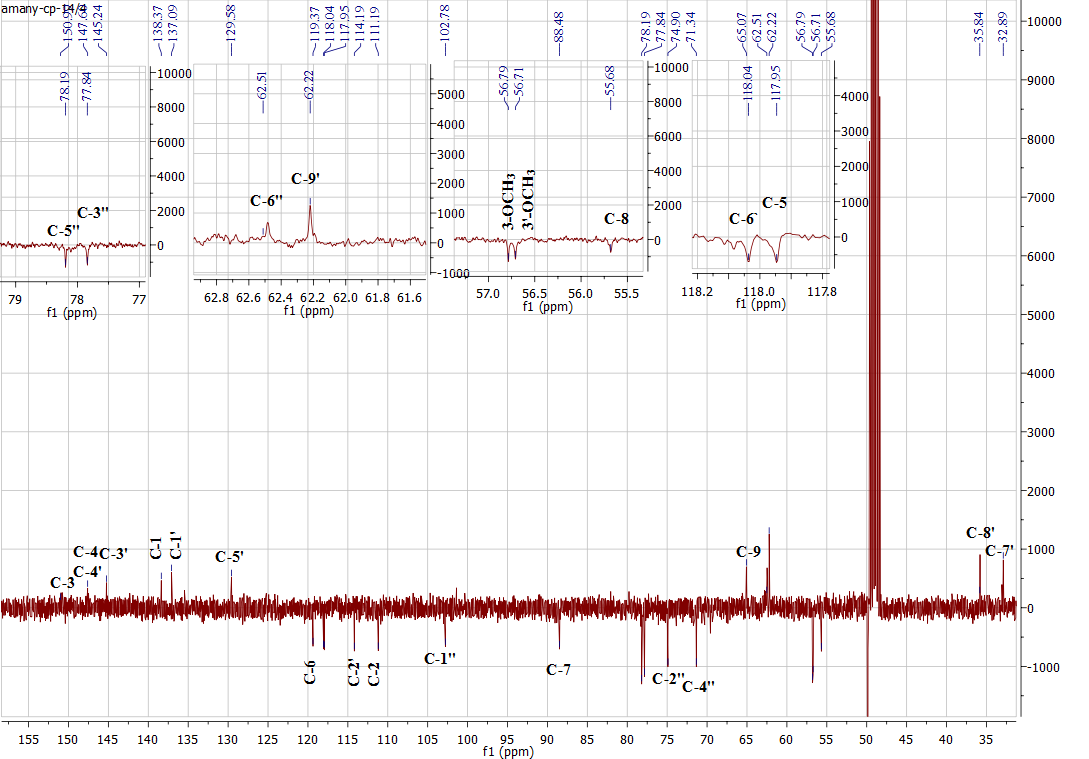
**

**Fig. S5c:** APT spectrum of dihydrodehydrodiconiferyl alcohol 4-*O*-*β*-D-glucoside (**6**)

**
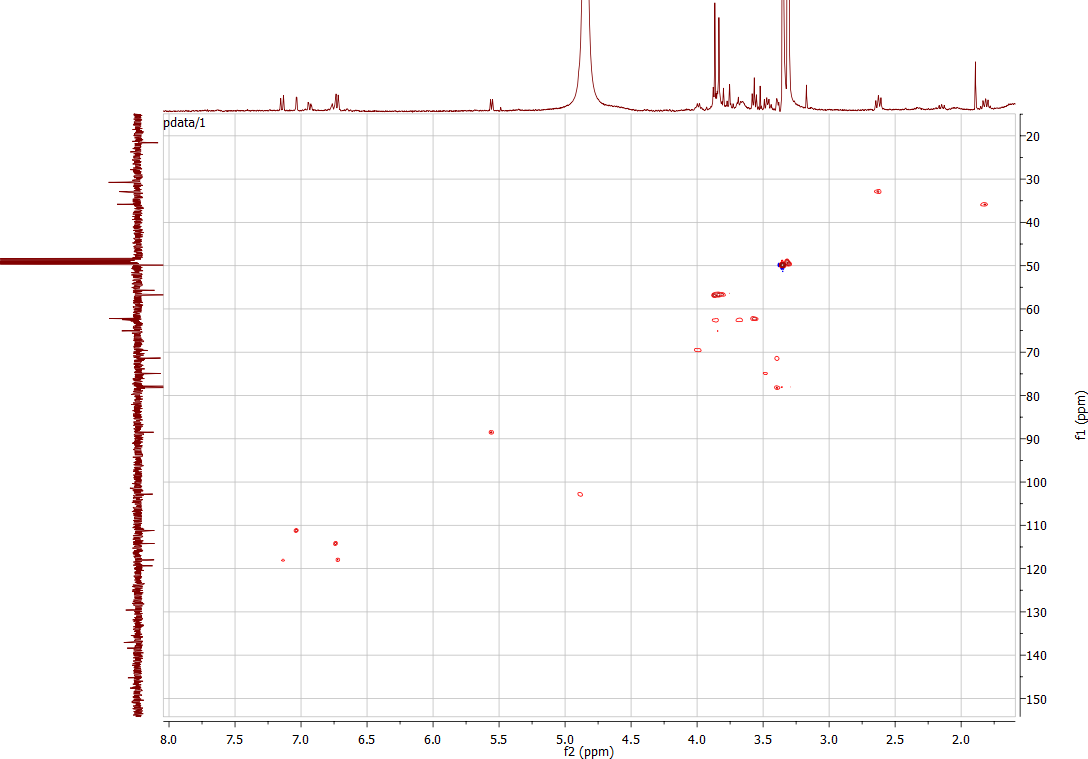
**

**Fig. S5d:** HSQC spectrum of dihydrodehydrodiconiferyl alcohol 4-*O*-*β*-D-glucoside (**6**)

**
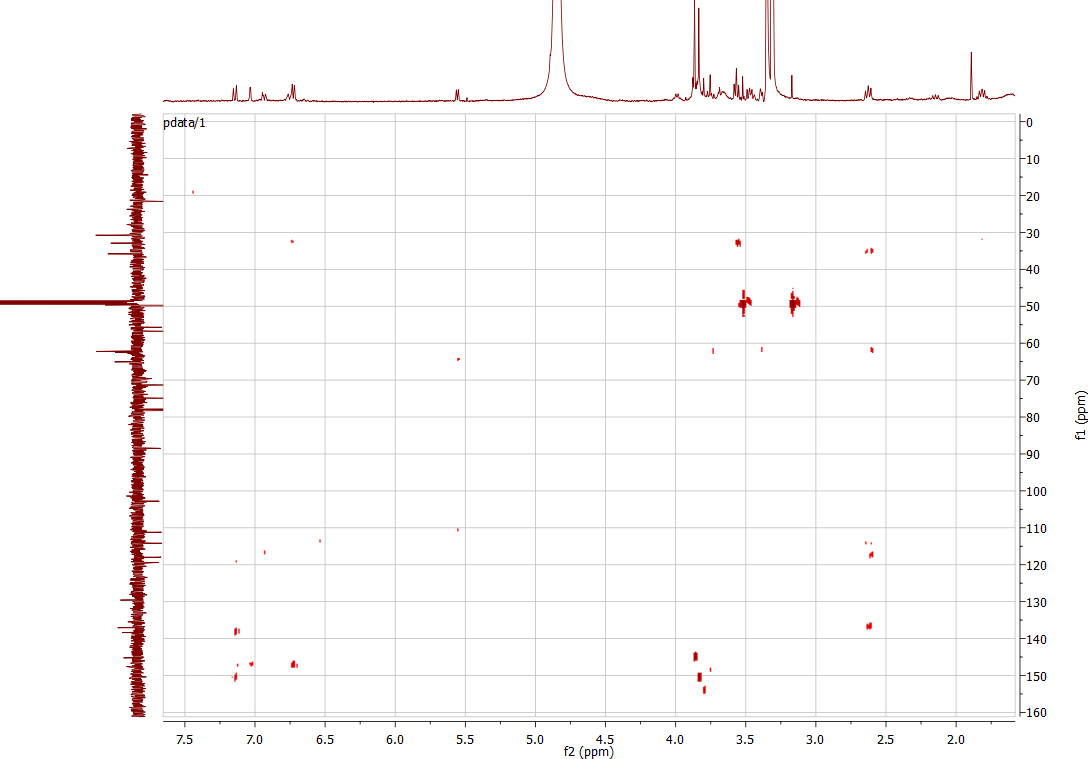
**


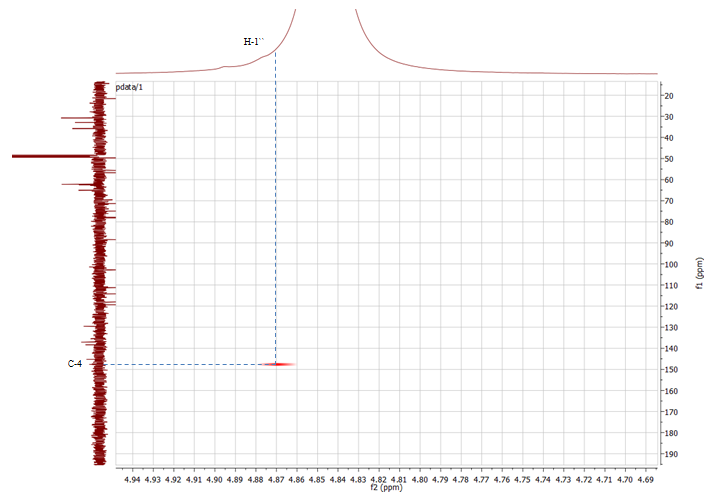


**Fig. S5e:** HMBC spectrum of dihydrodehydrodiconiferyl alcohol 4-*O*-*β*-D-glucoside (**6**)


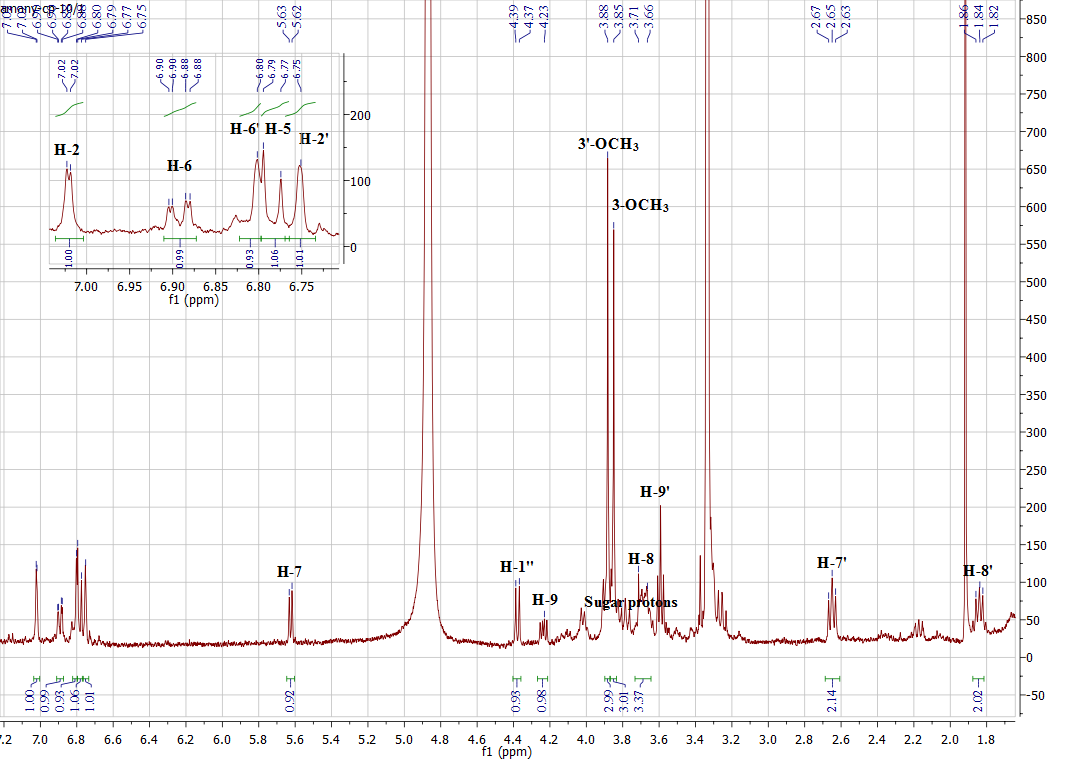


**Fig. S6a:** ^1^H NMR spectrum of dihydrodehydrodiconiferyl alcohol 9-*O*-*β*-D-glucoside (**7**)

**
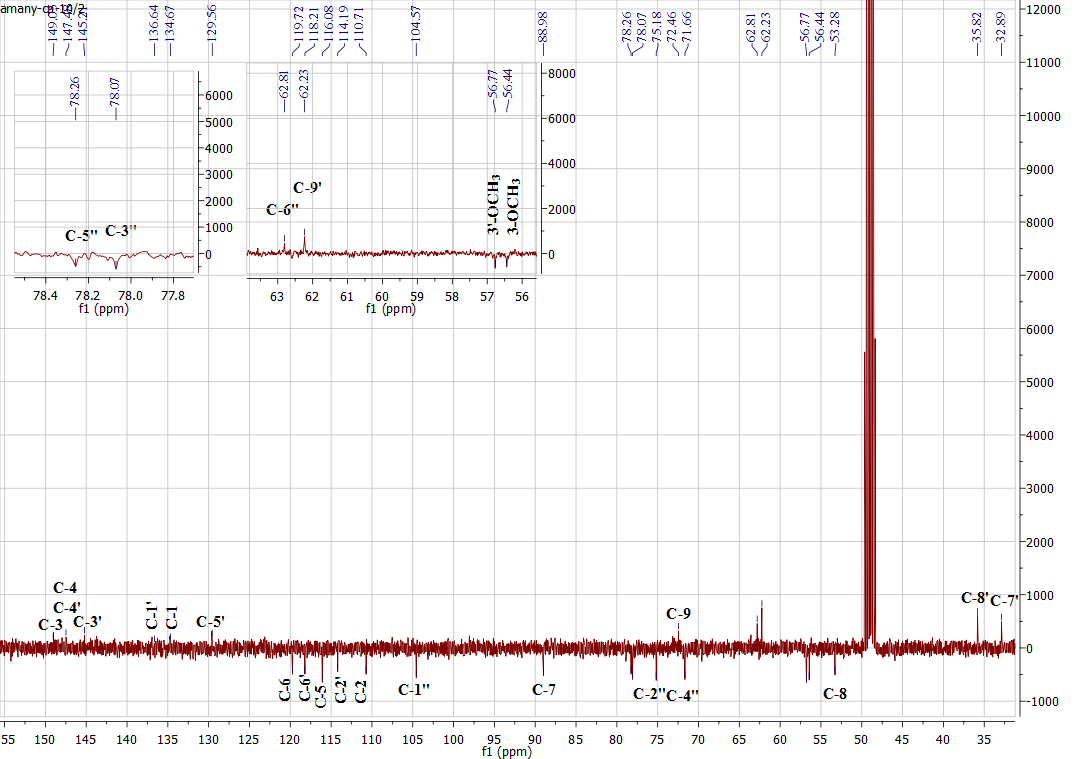
**

**Fig. S6b:** APT spectrum of dihydrodehydrodiconiferyl alcohol 9-*O*-*β*-D-glucoside (**7**)

**
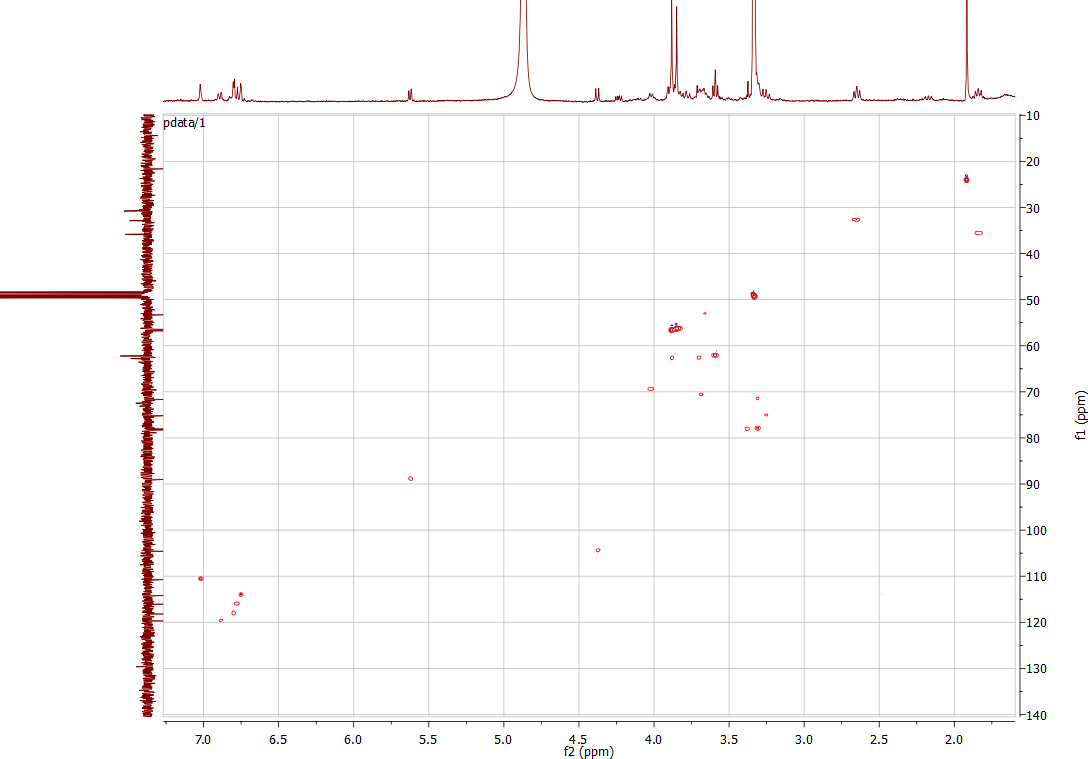
**

**Fig. S6c:** HSQC spectrum of dihydrodehydrodiconiferyl alcohol 9-*O*-*β*-D-glucoside (**7**)

**
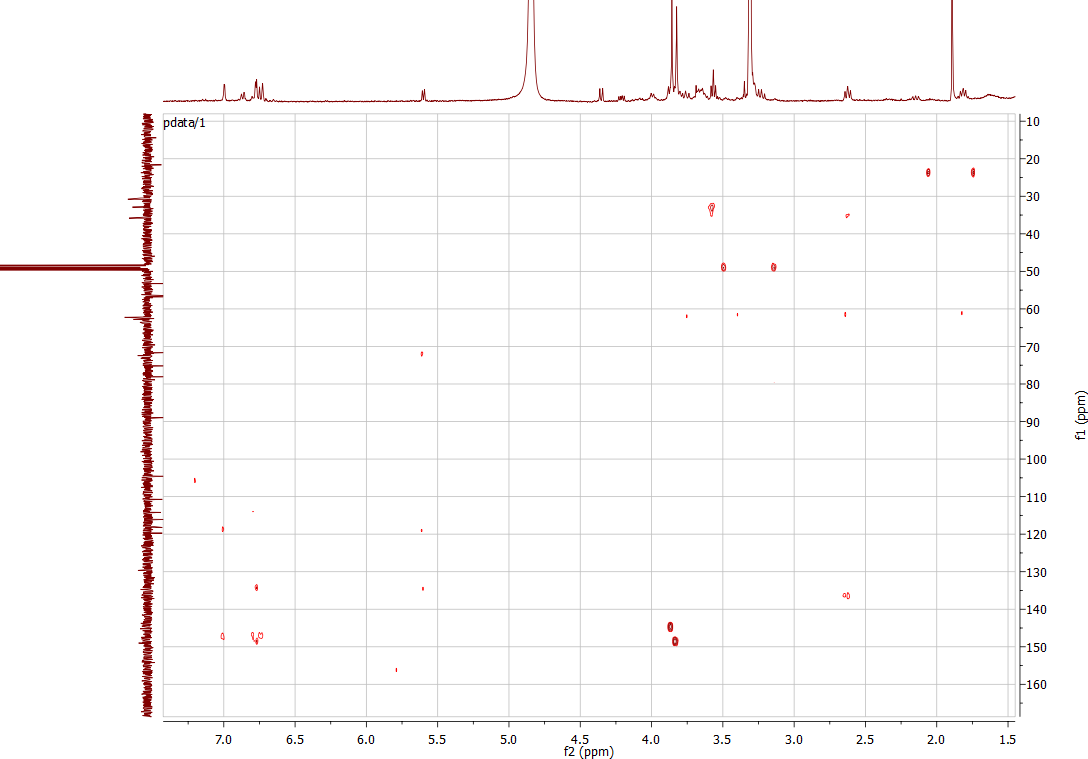
**
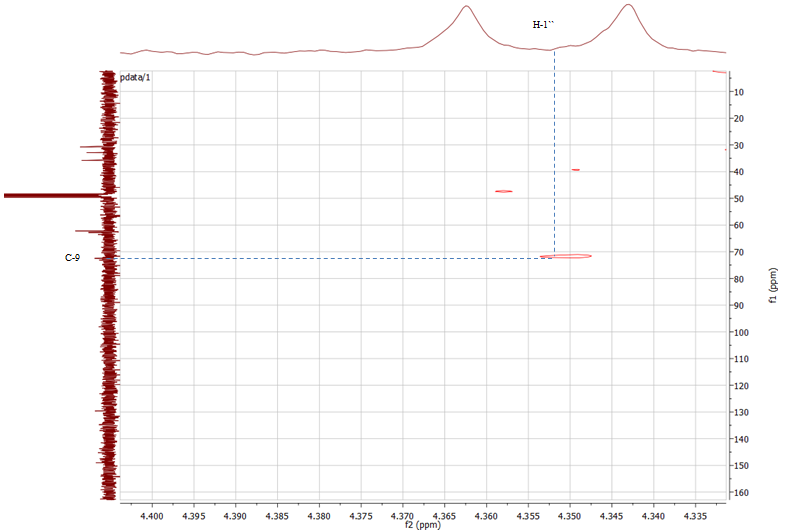


**Fig. S6d:** HMBC spectrum of dihydrodehydrodiconiferyl alcohol 9-*O*-*β*-D-glucoside (**7**)
